# Surface runoff alters cave microbial community structure and function

**DOI:** 10.1371/journal.pone.0232742

**Published:** 2020-05-06

**Authors:** Madison C. Davis, Maria A. Messina, Giuseppe Nicolosi, Salvatore Petralia, Melvin D. Baker, Christiana K. S. Mayne, Chelsea M. Dinon, Christina J. Moss, Bogdan P. Onac, James R. Garey

**Affiliations:** 1 Department of Cell Biology, Microbiology and Molecular Biology, University of South Florida, Tampa, FL, United States of America; 2 Centro Speleologico Etneo, Catania, Italy; 3 Department of Life Sciences and Systems Biology, University of Turin, Turin, Italy; 4 Applied Chemical Works, Paternò (Ct), Italy; 5 Karst Research Group, School of Geosciences, University of South Florida, Tampa, FL, United States of America; Universita degli Studi di Milano-Bicocca, ITALY

## Abstract

Caves formed by sulfuric acid dissolution have been identified worldwide. These caves can host diverse microbial communities that are responsible for speleogenesis and speleothem formation. It is not well understood how microbial communities change in response to surface water entering caves. Illumina 16S rRNA sequencing and bioinformatic tools were used to determine the impact of surface water on the microbial community diversity and function within a spring pool found deep in the Monte Conca Cave system in Sicily, Italy. Sulfur oxidizers comprised more than 90% of the microbial community during the dry season and were replaced by potential anthropogenic contaminants such as *Escherichia* and *Lysinibacillus* species after heavy rains. One sampling date appeared to show a transition between the wet and dry seasons when potential anthropogenic contaminants (67.3%), sulfur-oxidizing bacteria (13.6%), and nitrogen-fixing bacteria (6.5%) were all present within the spring pool.

## Introduction

A number of caves worldwide are now recognized to be the result of a process often identified as sulfuric acid speleogenesis (SAS) [see 1 and references therein]. This process was first proposed by Principi [2] and is used to describe the formation of caves via dissolution of limestone by sulfidic groundwaters. Among the most well-known and investigated SAS caves are those from Guadalupe Mountains, USA [3, 4], Movile, Romania [5], Frasassi, Italy [6, 7], Cueva de Villa Luz, Mexico [8, 9], and Lower Kane Cave, USA [10, 11].

Microbial communities in caves play a role in precipitation of speleothems, such as pool fingers [12, 13], moonmilk [12, 14], snottites [8, 15], and appear to contribute to sulfuric acid speleogenesis [8, 11, 16, 17]. Cave microbial communities are often diverse and influenced by the cave environment [18, 19]. Soil bacteria may be brought into caves from surface water inputs [20], whereas the presence of fecal coliforms may be caused by anthropogenic contamination and/or bat guano deposits [21, 22].

Cave microbes can have diverse metabolic functions [18]. Those involved in sulfur cycling have been identified in several caves, including Cesspool [16], Frasassi [17, 23, 24], Movile [5, 25], and Lower Kane [26]. Carbon fixation [26], nitrogen cycling, and methane cycling [24, 25] appear to be important microbial processes within caves. Iron and manganese deposits found in caves have been attributed to the presence of microbial iron and manganese cycling [27, 28].

Monte Conca is a karst cave in Sicily, Italy that has a sulfidic spring within the inner part of the lower gallery. The hydrochemistry of this spring was investigated by Messina et al. [29], who suggested there could be an active sulfur-cycling microbial community. During heavy rains, large volumes of water (3–15 L/s) enters the cave and reach the spring pool. The present study focuses on identifying the impact of surface water entering the cave on the microbial community diversity and function of the Monte Conca spring pool. We hypothesize that surficial inputs are the primary drivers of seasonal change within the microbial community function and diversity within the spring pool.

## Materials and methods

### Site description

**Fig 1:**
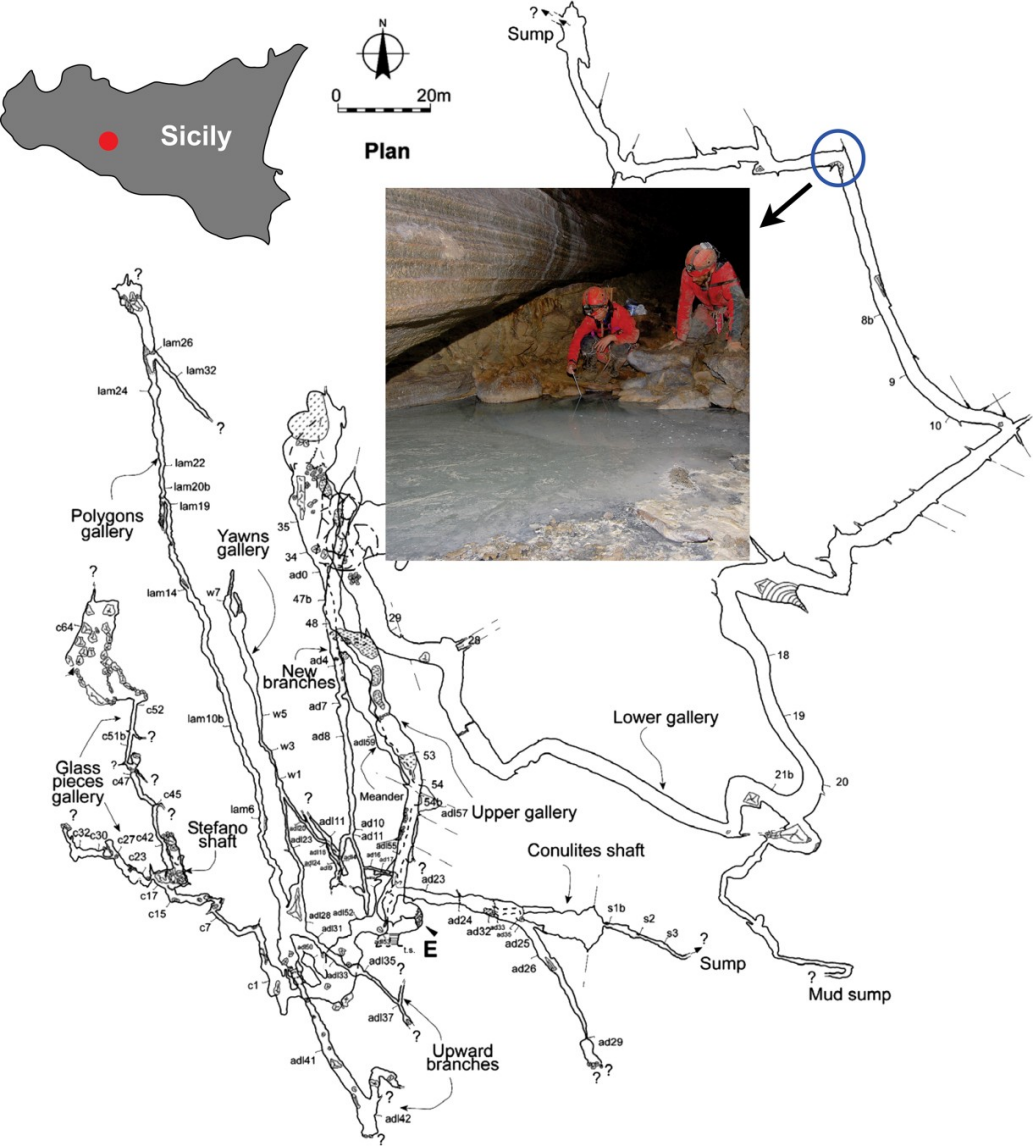
Map of the Monte Conca Cave system in Sicily, Italy. Map of Sicily showing the location of Monte Conca (red dot). Plan view map of the cave with indication of its entrance (E) and location of the sulfidic spring (blue circle) republished from [31] under a CC BY license, with permission from the author, original copyright 2008. Photo of the team sampling the water at the sulfidic spring pool. Note the stream discharging the pool in the foreground.

Monte Conca (37°29’23”N—13°42’49”E) is the first reported gypsum cave with an active sulfidic spring [29]. The cave develops in upper Miocene (Messinian) evaporites and is the longest and deepest gypsum karst system in Sicily (Fig 1). Madonia and Vattano [30] provide the most recent description of the cave’s genesis. A sulfidic spring is present within the inner part of the lower gallery year-around (Fig 1). This spring creates a small pool, which changes depth according to seasons. After heavy rains, surface water can reach the spring pool.

#### Sampling strategies

Water samples were collected from the sulfidic spring pool for biological and hydrochemical analyses on the following days: July 11, 2015; August 29, 2015; February 6, 2016; and December 10, 2016. July and August samples reflect the dry season, whereas the sample from February is typical for the wet season. Sampling was not possible during much of the wet season due to dangerous conditions resulting from high water levels within the cave. The December sampling is considered as a transition period between the wet and dry seasons because the rainfall amount could not be classified as wet or dry season.

Five replicate water samples were collected using gloves and stored at 5°C for each of the following: sulfide, total organic carbon (TOC), sulfate, and biological analysis. Replicates for sulfide were stabilized with 1.5 mL of zinc acetate (9 g/30 mL) and stored at room temperature until UV-Vis spectrophotometric analysis. Total organic carbon samples were acidified at pH<2 with phosphoric acid and stored in dark glass bottles. All bottles filtration apparatuses, and filters were sterilized by UV treatment for 1 hour at 254 nm.

### Hydrochemical analyses

The following measurements were performed *in situ*: pH (Carlo ERBA pH-meter), air temperature (HOBOware sensor), water conductivity, and temperature (CM-35 Crison conductometer). Sulfate and TOC were measured using Thermo Scientific Dionex ion chromatography and Hach instruments, following the UNI EN ISO 10304–1:2009 and UNI EN 1484:1999 procedures, respectively. Sulfide concentrations were measured by Cline’s [32] methylene blue method.

Statistical analyses of the replicate hydrochemical data were performed using Primer v7/Permanova+ statistical software (Primer-E Ltd., Albany, New Zealand). Hydrochemical data were transformed (log X+1), normalized (subtracted the mean across all samples and divided by the standard deviation of the variable), and clustered using Euclidean distance before visualizing with principal coordinate analysis (PCoA, Primer v7/Permanova+).

### Biological analyses

Water samples (500 mL) were filtered through sterile 0.22-μm filters (Isopore, Ireland). Filters were shipped frozen and on ice to the lab in sterile 6 cm Petri dishes for DNA extraction. Environmental DNA was extracted aseptically from the filters using the PowerSoil kit (Qiagen, USA). Preliminary length heterogeneity polymerase chain reaction (LH-PCR) analyses were carried out as described by Menning et al. [33], who profiled microbial communities utilizing the V4 region of the 16S rRNA gene in bacteria. These preliminary measurements [34] were used to determine the variability of the microbial communities at different sampling locations within the spring.

A year-long study using 16S Illumina 300-bp paired end sequencing on three replicate DNA samples from each date. Gene sequencing was carried out by PCR amplification of the V4 region with pro341f and pro851r primers [35] adapted for Illumina MiSeq sequencing by Applied Biological Materials, Inc (Richmond, BC and subsequent purification with AmPure XP beads (Illumina, San Diego, California, USA). Mothur software [36] was used to assemble paired-end reads and to remove sequences that were ambiguous or greater than the expected length. Chimeras were eliminated using the VSEARCH algorithm [37] in mothur. Sequences were aligned in mothur using the Silva Version 128 database. Operational taxonomic units (OTUs, ≥97% similarity) were clustered using the OptiClust algorithm [38].

Microbial community structure and statistical analyses of the replicate sequence data were analyzed using Primer v7/Permanova+ statistical software. Square-root transformation and clustering using Bray-Curtis similarity were utilized for the top 2000 OTUs for Bacteria before analyzing with PCoA (Primer v7/Permanova+). A Bio-Env (BEST) analysis was performed to determine the relationships between the biological and abiotic data. Rarefaction curves were produced using Mothur [36]. Diversity indices were calculated for each replicate separately for all sequences excluding singletons, using EstimateS software (EstimateS 9.1.0). Evenness was calculated by dividing the mean Shannon diversity by the natural log of the total number of OTUs of each replicate.

The 100 most abundant Bacterial OTUs (referred herein as the top 100) were used in our functional analysis to ensure over 80% of the sequence abundance for each date was analyzed. A total of 342 Bacterial OTUs were investigated due to overlap of OTUs between dates. A representative sequence from each OTU was used as a Genbank query for provisional identification, and those that could not be identified were called “unidentified”. OTUs with the same provisional identification were combined for subsequent analyses.

The potential metabolic function of each OTU was assigned by a review of the literature for each identified prokaryote. Predictive functional profiling may not accurately characterize the extremophiles within caves due to high variability of some gene families [39] that may be present within the cave microbiome. Obligate anaerobes and obligate or microoxic aerobes were classified separately than facultative bacteria. Halotolerant and halophilic microbes were categorized together. Sulfur reducers included sulfur disproportionation and dissimilatory sulfate reduction, whereas sulfur oxidizers comprised microbes that may oxidize any sulfur compounds. Nitrogen reducers included microbes that carry out denitrification, nitrogen fixation, and dissimilatory nitrogen reduction. Nitrogen oxidizers consisted of microbes that can utilize nitrification and anaerobic ammonia oxidation. Though not technically a function, the term “anthropogenic microbes” was used to describe microbes from potential contaminants that may entered the cave system and could therefore affect the endemic community’s function.

The relative abundance of sequences with metabolic functions (referred to herein as sequence abundance) was calculated independently by each date. The number of sequences with a provisional function was divided by the total number of sequences in each date and converted to a percent. The 342 Bacterial OTUs that represent the 100 most abundant Bacterial sequences for each sampling date were analyzed for function and represent over 92% of the sequences in our dataset.

## Results

Conductivity, sulfate, hydrogen sulfide, and rainfall measurements were significantly different between samples collected in the wet and dry seasons (Table 1). Conductivity was higher (3.81 mS/cm) at the transition between wet and dry seasons, whereas sulfate was lowest (1831 mg/L) during this time. TOC values for the transition period (4 mg/L) were between the wet season (2 mg/L) and the dry season (5–7 mg/L). Hydrogen sulfide concentrations during the dry season (10–14 ppm) are more similar to the transition period (8.5 ppm) compared to the wet season (3 ppm). The lowest temperature of the spring water was recorded during the wet season (13°C).

**Table 1:** Meteorological, physical, and hydro-chemical data for Monte Conca spring.

Date Parameters	July 11, 2015 Dry	August 29, 2015 Dry	February 6, 2016 Wet	December 10, 2016 Transition
**Rainfall (mm) (previous month)**	37	18	117	56
**Number of rainy days (previous month)**	11	5	21	19
**Conductivity (mS/cm)**	3.37	3.21	2.83	3.81*
**TOC (mg/L)**	5	7	2	4*
**Sulfate (mg/L)**	2210	2114	1886	1831*
**Hydrogen sulfide (ppm)**	10	14	3	9*
**Cave water temperature (ºC)**	15	15	11	14*
**Spring pool water temperature (ºC)**	17	17	13	15*
**Cave air temperature (ºC)**	16	16	11	16*
**Air temperature (ºC) at the spring site**	12	17	15	17*

* denotes average of five replicate measurements

**Fig 2:**
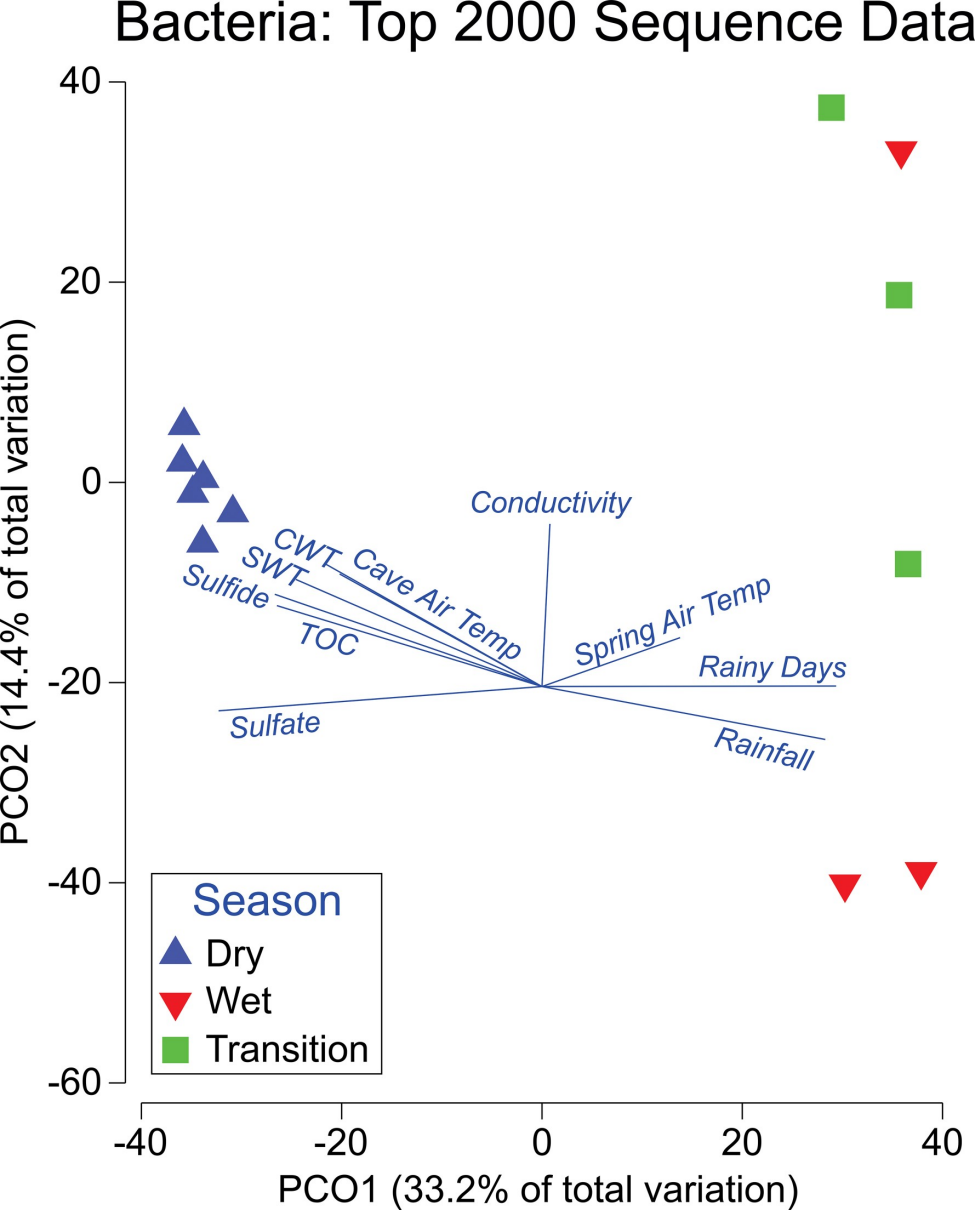
Principal coordinate analysis (PCoA) of the top 2000 OTUs. Each data point represents a replicate sample of bacterial communities, depicting thousands of 16S rRNA sequences from the data in S2 Table. The three replicates from each date are shown and labeled by season. The hydrochemical information from Table 1 is incorporated in this PCoA plot.

Mothur analysis of Illumina sequencing revealed a total of 353,008 sequences represented by 16,381 Bacterial OTUs. Illumina sequences from the 2000 most abundant bacterial OTUs encompassed over 97% of the sequence abundance and were analyzed with PCoA and coded by season (Fig 2). Each point on this PCoA represents a replicate that contains thousands of sequences from the top 2000 OTUs from the entire dataset. Both axes together account for 48.7% of the total variation within these samples. The lines on Fig 2 correspond to a correlational analysis of the hydrochemical results to the sequence data. Sequences from the wet season clustered separately from the dry season. The transition is different from the other two seasons (p = 0.001) but appears more similar to the wet one.

**Fig 3:**
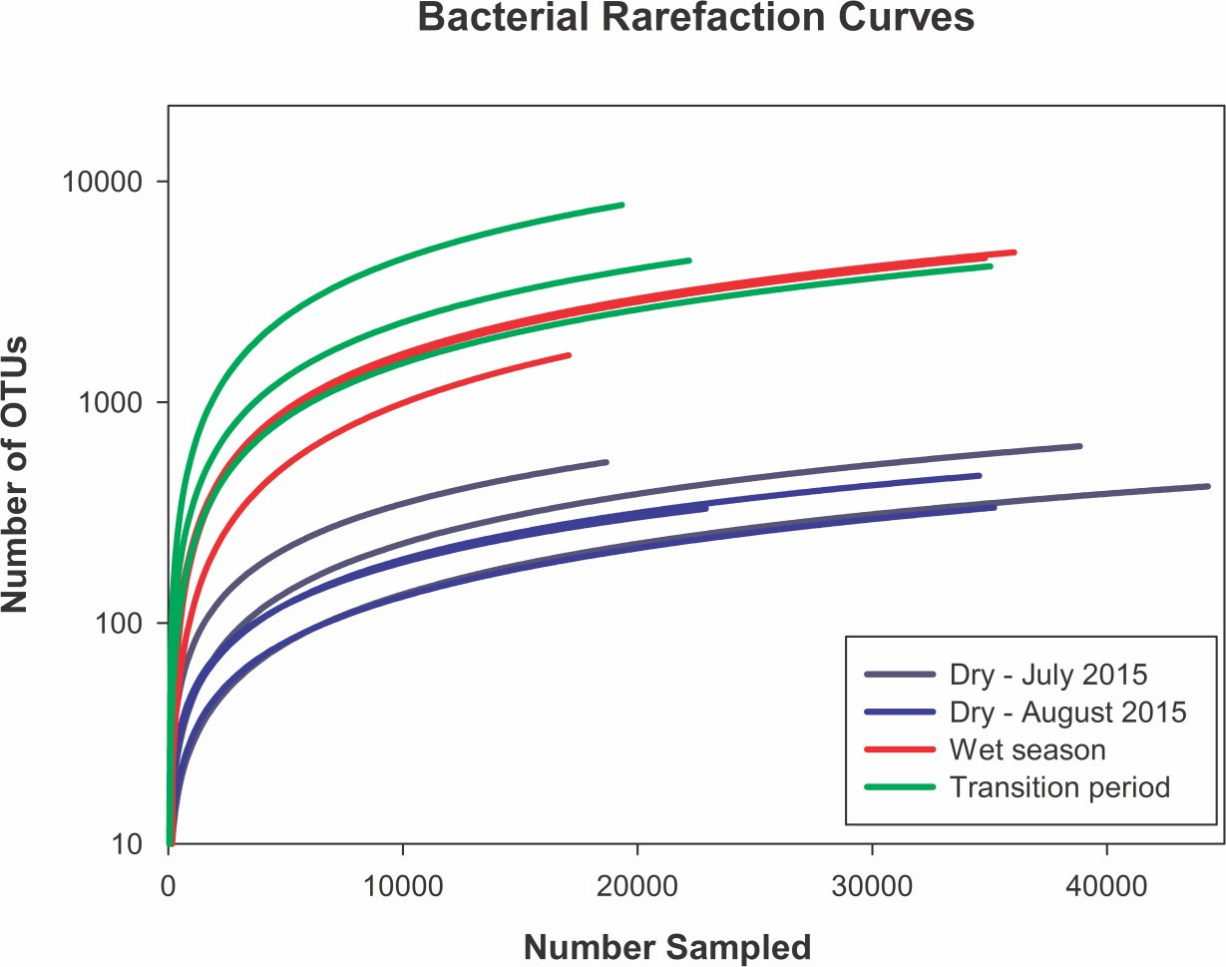
Microbial diversity of the Monte Conca spring pool. Bacterial rarefaction curves of the triplicates from each season/period.

Bioinformatic results are shown in S1 Table. These include the percent match to closest identified genus and the known metabolic functions of the provisionally identified genus. The relative abundance of each OTU from the triplicate samples are shown in S2 Table. The rarefaction curves (Fig 3) are used here to indicate the completeness of the microbiome sequencing.

Identity and function of the top 100 Bacterial OTUs in each date were analyzed in detail and represent 326,479 sequences. Excluding singletons, the 100 most abundant OTUs account for ~95% of the sequences within the dataset. Despite a 97% cut-off for OTU clustering, some OTUs have the same provisional identification, which were combined together under the same provisional identification. The 10 most abundant provisionally-identified bacterial taxa from each date are shown in Table 2.

**Table 2:** The abundance (abund.) and relative abundance (relative abund.) of the 10 most common bacterial taxa for each sampling date. Relative abundance was calculated by dividing the sequence abundance of each taxon by the total number of sequences for the sample date.

Dry season							
July 11, 2015				August 29, 2015			
Provisional identification	Num. OTUs	Abund.	Relative abund.	Provisional identification	Num. OTUs	Abund.	Relative abund.
Thiovirga	12	72157	92.91%	Sulfurovum	17	52013	41.26%
Sulfurimonas	5	13188	16.98%	Thiovirga	18	22775	18.07%
Sulfurovum	9	5086	6.55%	Sulfurimonas	5	4512	3.58%
Arcobacter	3	3605	4.64%	Thiomicrospira	4	4496	3.57%
Unidentified	7	1526	1.96%	Arcobacter	1	3168	2.51%
Escherichia	1	1175	1.51%	Escherichia	2	2197	1.74%
Sulfurospirillum	4	712	0.92%	Sulfurospirillum	4	613	0.49%
Sulfuricurvum	2	648	0.83%	Unidentified	13	386	0.31%
Bacillus	5	249	0.32%	Lysinibacillus	1	329	0.26%
Thiomicrospira	2	201	0.26%	Paludibacter	4	251	0.20%
Wet season				Transition			
February 6, 2016				December 10, 2016			
Provisional identification	Num. OTUs	Abund.	Relative abund.	Provisional identification	Num. OTUs	Abund.	Relative abund.
Escherichia	71	64501	81.79%	Escherichia	23	34595	54.66%
Lysinibacillus	18	7358	9.33%	Unidentified	18	5826	9.20%
Phyllobacterium	3	85	0.11%	Lysinibacillus	11	3551	5.61%
Mycoplasma	2	43	0.05%	Sulfurimonas	16	2941	4.65%
Afipia	1	28	0.04%	Thiovirga	13	2671	4.22%
Sphingopyxis	1	21	0.03%	Sulfurovum	4	886	1.40%
Unidentified	1	16	0.02%	Thiothrix	4	316	0.50%
Bdellovibrio	1	16	0.02%	Phaeocystidibacter	2	179	0.28%
Pseudomonas	1	16	0.02%	Arcobacter	3	155	0.24%
Shigella	1	16	0.02%	Sulfurospirillum	1	76	0.12%

An abbreviated potential metabolic function of the microbial communities is illustrated in Table 3. Roughly 90% of the bacteria sequence abundance in the wet season was identified as anthropogenic microbes (Table 3). Sulfur oxidizers were present in the dry season (90.5–94.9%) and in the transition period (11.4%). Denitrification (6.8%) appears in the transition period. Nitrogen fixers were identified in the dry season (3.8%) and in the transition period (6.5%), but not in the wet season (0.0%). The percentage of microbial community function does not equal 100% due to overlap in taxa that may perform more than one function.

**Table 3:** The percent abundance of the potential metabolic functions.

	Potential Bacterial Function (%)			
date	7/11/2015	8/29/2015	2/6/2016	12/10/2016
season	dry	dry	wet	transition
anthropogenic	3.6	3.7	89.5	67.3
sulfur oxidizer	94.9	90.5	0.0	13.6
denitrifier	0.2	0.1	0.0	6.8
nitrogen fixation	3.8	3.8	0.0	6.5

The diversity indices for each date are shown in Table 4.

**Table 4:** The average microbial diversity of each sampling date.

Microbial diversity indices				
Date	7/11/2015	8/29/2015	2/6/2016	12/10/2016
**Average of total OTUs**	289	228	626	1017
**Shannon Mean**	1.14 ± 0.43	1.70 ± 0.22	1.82 ± 1.08	3.77 ± 0.17
**Evenness**	0.20 ± 0.07	0.31 ± 0.03	0.28 ± 0.14	0.54 ± 0.17

## Discussion

### Wet season

**Fig 4:**
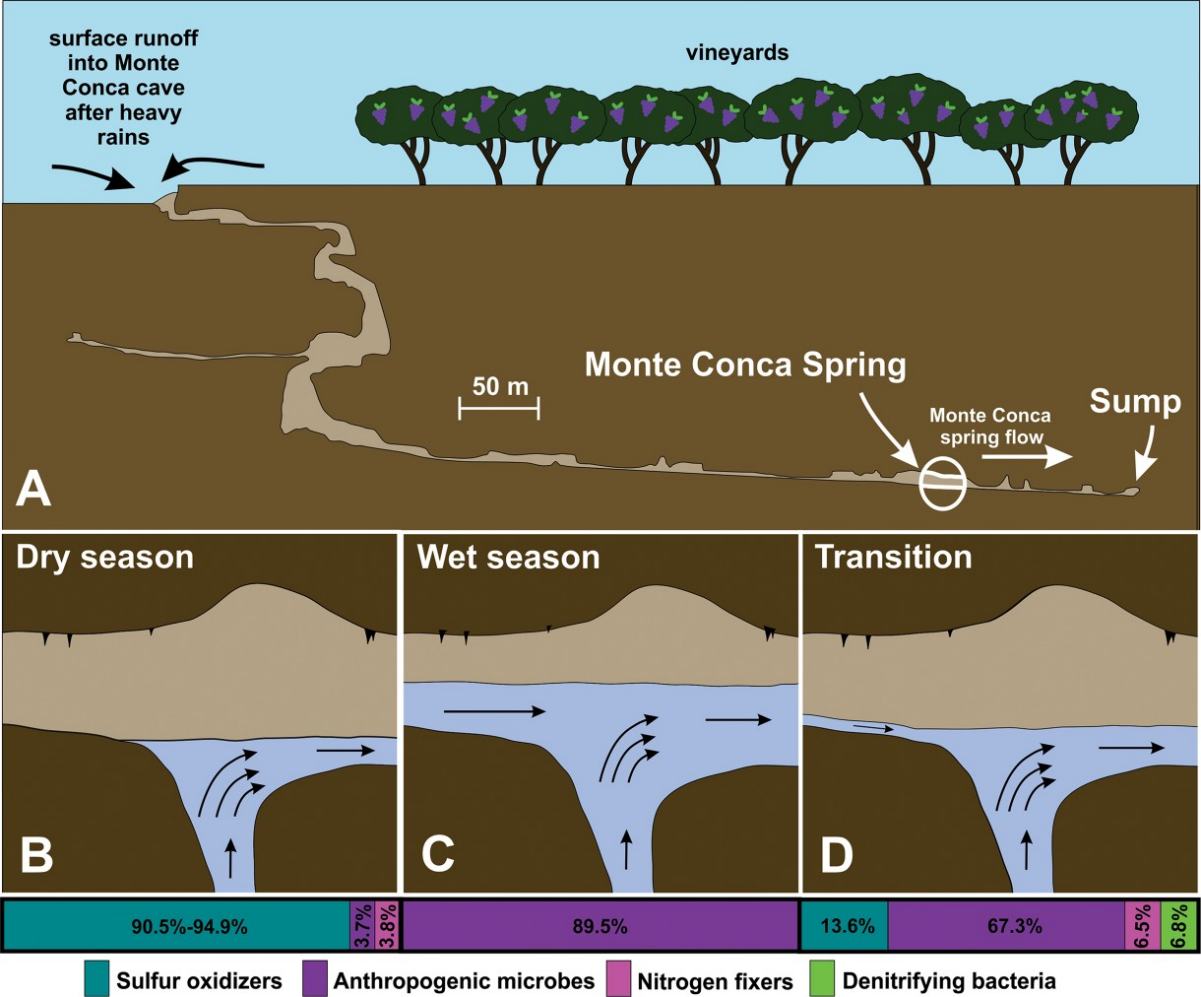
The seasonal relationship between surface runoff and sulfidic spring in Monte Conca Cave. (A) Profile view of the Monte Conca Cave. Hydrogen sulfide is produced in deeper parts of the gypsum karst and is discharged into the spring pool (circled), which generates a small stream that flows northward towards the sump (see Fig 1). After heavy rains, surface water enters the cave and reaches the lower gallery flowing northward towards the sump. (B-D) The hydrological settings at the sulfidic spring during different periods. The relative microbial abundance is shown in bar graphs for each of the three seasons to demonstrate community changes over time with the abundances from Table 3.

During the wet season, lasting from January until May, water runoff from the surface enters the Monte Conca Cave [29]. Spring water temperature, conductivity, TOC, sulfate, and hydrogen sulfide concentrations were all lower in the wet season compared to the dry season, whereas the microbial diversity was similar to the dry season (Table 1, Table 4). Microbes identified as potential anthropogenic contaminants, such as *Escherichia* and *Lysinibacillus* comprise 89.5% and 3.7% of the sequences within the wet and dry season, respectively (Table 2, Fig 4). The abundance of these microbes during the wet season suggests that surface runoff introduces them into the cave, and their dominance of over 90% of the community may explain the low evenness values found during this season (Table 4).

The presence of *Escherichia* has been documented outside cave entrances [40] and in caves [e.g. 41, 42]. Surface water can be contaminated by a number of mechanisms [43, 44] and can support *Escherichia* for several days [45]. Once inside the cave, contaminated water may flow along karst conduits for several kilometers allowing for large portions of the cave to become contaminated with fecal microbes [46]. Sources of contamination, the storage capacity of bacteria in soil and water, and the bacterial survival rate in groundwater are responsible for seasonal variations of bacterial contaminants in caves [47]. Since enterobacteria are known to survive in soils [45], the *Escherichia* in Monte Conca are likely derived from the surrounding soils, particularly from the agricultural terrains above the cave. The presence of *Lysinibacillus*, a common soil microbe [48, 49], also supports this hypothesis. Some *Lysinibacillus* species are pathogenic and/or can be found in farming soil (see S1 Table), thus this genus could be considered “anthropogenic” for the purposes of this study. The presence of soil and enteric bacteria identified in Monte Conca are consistent with other subsurface studies [e.g. 41, 42, 46, 50]. Molecular-grade water processed through each step in the biological analyses process did not yielded these genera, therefore it is unlikely that these communities are the result of process contamination.

### Dry season

During the dry season, which lasts from June through December, the Monte Conca spring pool has a different microbial community compared to the wet season (Fig 4). Communities in the dry season had low diversity and evenness (Table 4), likely because fewer taxa are dominant during this period. Although Messina et al. [29] suggested that *Acidithiobacillus* and *Beggiatoa* could be responsible for sulfur oxidation within this cave, this study identified *Sulfurovum*, *Sulfurimonas*, *Thiovirga*, and *Arcobacter* in the Monte Conca spring pool (Table 3). These genera have been found in Movile Cave and the Frasassi cave system [16, 17], but other sulfur-oxidizers have been documented from many other cave environments [16, 23, 24, 50]. Decreases in pH of the sulfidic spring during the dry season [29] may be attributed to these microbes. Similar sulfidic environments with high concentrations of carbon [51] and low levels of oxygen [52] have been shown to host sulfur oxidizers. Denitrifiers are known to be inhibited by high concentrations of hydrogen sulfide [53, 54], which may explain why they are not abundant in the dry season community.

### Transition between wet and dry seasons

Large volumes of water entering the Monte Conca Cave (3–5 L/s) can create dangerous situations that limit access to the spring pool during the wet season. The December 2016 sampling was carried out after heavy rainfall, originally to be included as a wet season sample. Rainfall during this period (56 mm) was approximately half of the typical wet season (117 mm) and greater than the dry season (18–37 mm), so these samples were designated as representing a transition between the wet and dry seasons.

The microbial analysis identified microbes that are common to both the wet and dry seasons (Table 2). Sulfur oxidizers and anthropogenic microbes recognized in the dry and wet seasons, respectively, were present within this sampling date. Anthropogenic microbes at this sampling date (67.3%) were less abundant than the wet season (89.5%), but richer than the dry season (3.6–3.7%). Microbes with the ability to fix nitrogen were higher during the transition period (6.5%) compare to the dry season (3.8%), and absent in the wet season. Microbes with the potential for denitrification were only identified within the December 2016 sample (6.8%).

### The ecosystem

Surface water inputs greatly affect the Monte Conca Cave environment. Examination of the bacterial PCoA analysis in Monte Conca Cave demonstrates different wet/dry season microbial communities (Fig 2). A chemolithoautotrophic community is present during the dry season months until it is replaced by anthropogenic microbes likely derived from surface-runoff during the wet season. The transition period between the seasons had the greatest microbial diversity (Table 3). According to the BEST analysis, rainfall (*p*_*s*_ = 0.578) accounted for the greatest variance within the microbial community, demonstrating the seasonal impact of surficial inputs into the cave system. The flooding events prior to the transition period and wet season could explain the diversity during these respective intervals (Table 3).

Hydrogen sulfide (Table 1) is likely produced underneath the Monte Conca Cave by gypsum reduction and is discharged into the spring pool (Fig 4). Although hydrogen sulfide is present within the cave year-round (Table 1), its highest concentration occurs in the dry season (10–14 ppm). Dry season microbial communities are dominated by sulfur-oxidizing bacteria (Table 3) due to the sulfidic spring conditions and from access to oxygen in the cave. Surface runoff into the cave disrupts these communities and may dilute the hydrogen sulfide (Table 1) in the spring pool.

Surface runoff can affect cave microbial communities such as those found in the Monte Conca spring pool. Anthropogenic microbial contaminants originating from outside of the cave environment can replace endemic cave communities. We identified one sampling date that appears to show a transition between the dry and wet seasons, which was corroborated by an increase in bacterial diversity. The microbial community during this transition period was the most diverse and consisted of potential anthropogenic contaminants from the surface in addition to the sulfur oxidizers that were identified in the dry season. This study demonstrates the impact of surface runoff on the microbial community structure and function of endemic cave communities.

## Supporting information

S1 Table Microbial community function. Functional analysis of the bacterial OTUs.
(PDF)

S2 Table Microbial community assemblages. OTU abundance of bacteria in each sample.
(PDF)
