# Supplementary material for: Surface runoff alters cave microbial community structure and function
Source: PLoS One. 2020 May 6;15(5):e0232742. doi: 10.1371/journal.pone.0232742 (PMC7202643; doi:10.1371/journal.pone.0232742)
Supplement: S2 Table — OTU abundance of bacteria in each sample. (PDF) [file pone.0232742.s002.pdf]

OTU Number	7.11.15 Rep1	7.11.15 Rep2	7.11.15 Rep3	8.29.15 Rep1	8.29.15 Rep2	8.29.15 Rep3	2.6.16 Rep1	2.6.16 Rep2	2.6.16 Rep3	12.10.16 Rep1	12.10.16 Rep2	12.10.16 Rep3
1	376	754	45	442	1602	146	13670	0	15966	0	0	4306
2	23366	8645	38916	3847	4685	2136	1	0	0	0	0	0
3	1610	1516	682	21374	17524	12666	0	0	0	0	74	0
4	373	133	565	4554	3404	2852	2	3	0	0	332	0
5	7479	3384	1797	1016	1603	1077	0	0	0	0	0	0
6	0	0	0	0	0	0	0	0	0	16611	0	0
7	0	0	0	0	0	0	0	14407	0	0	0	1506
8	52	110	3	51	259	19	1463	0	1871	742	0	0
9	2342	653	516	1232	1123	813	0	0	0	0	0	0
10	33	24	88	1257	1685	1148	0	0	0	0	0	0
11	0	0	0	0	0	0	0	0	0	0	0	0
12	0	0	0	0	0	0	0	6155	0	0	0	0
13	0	0	0	0	0	0	0	0	5221	0	0	0
14	0	0	0	0	0	0	0	0	0	4405	0	0
15	209	475	307	7	84	58	0	0	0	0	0	0
16	5	3	0	1	1	7	0	0	0	0	0	0
17	0	0	0	0	0	0	0	0	0	0	0	2395
18	38	99	273	97	488	199	0	0	0	0	0	0
19	354	150	124	47	76	52	0	0	0	0	0	0
20	0	0	0	0	0	0	0	0	0	1825	241	0
21	0	0	0	0	0	0	0	0	0	0	0	0
22	0	0	0	0	0	0	0	2021	0	0	0	0
23	0	0	0	0	0	0	0	0	2018	0	0	0
24	6	5	2	432	279	172	0	0	0	0	0	0
25	0	0	0	0	0	0	0	1787	0	0	0	0
26	0	0	0	0	0	0	0	0	0	0	1782	0
27	616	421	110	21	36	31	0	0	0	0	0	0
28	0	0	0	0	0	0	0	0	0	1509	0	0
29	225	132	83	27	56	61	0	0	0	0	0	0
30	0	0	0	0	0	0	0	0	0	0	0	0
31	0	0	0	0	0	0	0	0	0	1309	0	0
32	35	34	25	56	108	262	0	0	0	0	0	0
33	0	0	0	0	0	0	0	1156	0	0	0	0
34	0	0	0	0	0	0	0	0	1151	0	0	0
35	27	8	21	83	195	108	0	0	0	0	0	0
36	0	0	0	0	0	0	0	0	0	0	922	0
37	272	143	43	12	47	27	0	0	0	0	0	0
38	0	0	0	0	0	0	0	0	0	0	0	0
39	0	0	0	0	0	0	0	0	0	0	0	0
40	197	126	41	7	18	13	0	0	0	0	0	0
41	0	0	0	0	0	0	0	0	0	0	0	700
42	0	0	0	0	0	0	0	0	0	0	668	0
43	0	0	0	0	0	0	0	0	0	0	0	664
44	2	2	0	5	143	103	0	0	0	0	0	0
45	0	0	0	0	0	0	0	0	640	0	0	0

OTU Number	7.11.15 Rep1	7.11.15 Rep2	7.11.15 Rep3	8.29.15 Rep1	8.29.15 Rep2	8.29.15 Rep3	2.6.16 Rep1	2.6.16 Rep2	2.6.16 Rep3	12.10.16 Rep1	12.10.16 Rep2	12.10.16 Rep3
46	0	0	0	0	0	0	0	0	0	0	0	0
47	7	30	2	2	3	2	0	0	0	0	0	0
48	0	0	0	0	0	0	0	0	0	0	595	0
49	0	0	0	0	0	0	0	578	0	0	0	0
50	0	0	0	0	0	0	0	0	0	0	0	540
51	0	0	0	0	0	0	0	0	0	0	504	0
52	0	0	0	0	0	0	0	0	0	0	0	483
53	5	3	5	19	21	6	0	0	0	0	0	0
54	1	7	4	18	84	74	0	0	0	0	0	0
55	0	0	0	0	0	0	0	0	0	0	0	0
56	0	0	0	0	0	0	0	0	0	0	0	401
57	0	0	0	0	0	0	0	0	0	0	0	0
58	0	0	0	0	0	0	0	0	0	0	0	385
59	0	0	0	0	0	0	0	0	0	0	0	0
60	12	5	12	5	3	7	0	0	0	0	0	0
61	0	0	0	0	0	0	0	0	0	0	352	0
62	0	0	0	0	0	0	0	0	0	0	0	0
63	0	0	0	0	0	0	0	0	0	0	0	318
64	5	4	1	2	13	1	28	0	0	0	0	0
65	0	0	0	0	0	0	0	0	0	0	0	0
66	0	0	0	0	0	0	0	298	0	0	0	0
67	0	0	0	0	0	0	0	0	0	0	0	296
68	0	0	0	0	0	0	0	0	0	0	289	0
69	0	0	0	0	0	0	0	0	0	0	0	274
70	0	0	0	0	0	0	0	0	0	272	0	0
71	0	0	0	0	0	0	0	0	0	0	0	268
72	0	0	0	0	0	0	0	0	0	0	265	0
73	57	67	14	8	9	7	0	0	0	0	0	0
74	0	0	0	0	0	0	0	0	0	0	0	0
75	0	0	0	0	0	0	0	0	260	0	0	0
76	0	0	0	0	0	0	0	0	0	251	0	0
77	0	0	0	0	0	0	0	0	0	0	248	0
78	11	9	0	0	1	0	0	0	0	0	0	0
79	0	0	0	2	3	2	0	0	0	0	0	0
80	12	5	5	11	13	53	0	0	0	0	0	0
81	0	0	0	0	0	0	0	0	225	0	0	0
82	0	0	0	0	0	0	0	0	0	0	0	0
83	3	4	0	3	2	2	28	0	0	0	0	0
84	0	0	0	0	0	0	0	0	0	0	0	218
85	0	0	0	0	0	0	0	218	0	0	0	0
86	0	0	0	0	0	0	0	0	0	0	0	0
87	0	0	0	0	0	0	0	0	212	0	0	0
88	0	0	0	0	0	0	0	0	0	207	0	0
89	1	204	0	0	0	2	0	0	0	0	0	0
90	0	0	0	0	0	0	0	202	0	0	0	0

OTU Number	7.11.15 Rep1	7.11.15 Rep2	7.11.15 Rep3	8.29.15 Rep1	8.29.15 Rep2	8.29.15 Rep3	2.6.16 Rep1	2.6.16 Rep2	2.6.16 Rep3	12.10.16 Rep1	12.10.16 Rep2	12.10.16 Rep3
91	0	0	0	0	0	0	0	0	0	0	193	0
92	0	6	0	1	21	19	0	0	0	0	0	0
93	0	0	0	0	0	0	0	0	0	192	0	0
94	0	0	0	0	0	0	0	0	0	0	0	191
95	0	0	0	0	0	0	0	0	0	0	0	0
96	0	0	0	0	0	0	0	0	0	0	0	0
97	0	0	0	0	0	0	0	0	0	0	0	177
98	34	12	7	2	11	0	0	0	0	0	0	0
99	0	0	0	0	0	0	0	0	0	0	0	174
100	22	12	14	2	4	0	0	0	0	0	0	0
101	0	0	0	0	0	0	0	0	0	0	0	173
102	0	0	0	0	0	0	0	0	0	0	171	0
103	0	3	0	2	4	33	0	0	0	0	0	0
104	0	0	0	0	0	0	0	163	0	0	0	0
105	0	0	0	0	0	0	0	0	155	0	0	0
106	0	0	0	0	0	0	0	0	152	0	0	0
107	0	0	0	0	0	0	0	0	0	0	0	150
108	1	0	0	2	1	0	26	0	0	0	0	0
109	0	131	0	0	0	0	0	0	0	0	0	0
110	0	0	0	0	0	0	0	141	0	0	0	0
111	1	0	0	3	45	16	0	0	0	0	0	0
112	6	101	3	0	0	0	0	0	0	0	0	0
113	0	0	0	0	0	0	0	0	136	0	0	0
114	0	0	0	0	0	0	0	0	0	0	0	0
115	0	0	0	0	0	0	0	0	0	0	131	0
116	0	0	0	0	0	0	0	0	0	0	0	131
117	0	0	0	0	65	4	0	0	0	0	0	0
118	4	4	2	1	5	3	0	0	0	0	0	0
119	11	9	3	1	15	10	0	0	0	0	0	0
120	0	0	0	0	0	0	0	0	0	0	125	0
121	6	0	19	20	30	30	0	0	0	0	0	0
122	0	0	0	0	0	0	0	0	0	0	0	119
123	25	26	6	2	7	8	0	0	0	0	0	0
124	0	0	0	0	0	0	0	0	0	0	0	0
125	0	0	0	0	0	0	0	0	0	0	0	112
126	0	0	0	0	0	0	0	0	0	0	0	111
127	0	0	0	0	0	0	0	0	111	0	0	0
128	0	0	0	0	0	0	0	0	0	0	0	110
129	0	0	0	0	0	0	0	0	0	0	0	109
130	1	3	0	6	2	2	2	0	0	0	0	0
131	1	1	1	0	1	0	16	0	0	0	0	0
132	0	0	0	0	0	0	0	0	0	104	0	0
133	0	0	0	0	0	0	0	0	0	0	103	0
134	0	0	0	0	0	0	0	0	0	102	0	0
135	0	1	3	10	27	44	0	0	0	0	0	0

OTU Number	7.11.15 Rep1	7.11.15 Rep2	7.11.15 Rep3	8.29.15 Rep1	8.29.15 Rep2	8.29.15 Rep3	2.6.16 Rep1	2.6.16 Rep2	2.6.16 Rep3	12.10.16 Rep1	12.10.16 Rep2	12.10.16 Rep3
136	0	0	2	29	36	29	0	0	0	0	0	0
137	0	0	0	0	0	0	0	0	0	0	0	0
138	0	0	0	0	0	0	0	0	0	0	0	0
139	1	0	0	20	12	3	0	0	0	0	0	0
140	0	0	0	0	0	0	0	0	0	0	97	0
141	1	4	0	1	1	0	0	0	0	0	0	0
142	0	0	0	0	0	0	0	0	0	0	0	94
143	0	0	1	26	17	47	0	0	0	0	0	0
144	0	0	0	0	0	0	0	0	0	0	93	0
145	21	15	6	6	9	1	0	0	0	0	0	0
146	0	0	0	0	0	0	0	0	0	0	91	0
147	0	0	0	0	0	0	0	0	0	0	90	0
148	5	0	15	18	15	19	0	0	0	0	0	0
149	0	0	0	0	0	0	0	0	0	87	0	0
150	0	1	0	0	13	33	0	0	0	0	0	0
151	0	0	0	0	0	0	0	0	0	0	0	86
152	0	2	0	0	0	0	7	0	0	0	0	0
153	0	0	0	0	1	2	6	0	0	0	0	0
154	36	18	6	0	1	0	0	0	0	0	0	0
155	0	0	0	0	0	0	0	0	0	0	0	0
156	1	3	0	0	1	0	13	0	0	0	0	0
157	0	0	0	0	0	0	0	0	0	0	0	81
158	0	0	1	1	1	3	0	0	0	0	0	0
159	2	1	3	8	12	44	0	0	0	0	0	0
160	18	27	5	1	3	4	0	0	0	0	0	0
161	6	53	0	0	0	0	0	0	0	0	0	0
162	0	0	0	0	0	0	0	0	0	0	0	77
163	0	0	0	0	0	0	0	0	0	0	0	77
164	0	0	0	0	0	0	0	0	0	0	77	0
165	0	0	0	0	0	0	0	0	0	0	76	0
166	0	0	0	0	0	0	0	0	0	0	0	76
167	0	1	0	0	0	0	0	0	0	0	0	0
168	0	0	0	0	0	0	0	0	0	0	0	0
169	0	0	0	0	0	0	0	0	0	0	0	68
170	0	0	0	0	0	0	0	0	0	0	67	0
171	0	0	0	0	0	0	0	0	0	0	66	0
172	0	0	0	0	0	0	0	0	0	0	65	0
173	0	0	0	0	0	0	0	0	0	65	0	0
174	0	0	0	0	0	0	0	0	0	0	64	0
175	0	0	0	0	0	0	0	0	0	0	64	0
176	1	2	1	1	7	0	0	0	0	0	0	0
177	0	0	0	0	0	0	0	0	0	0	62	0
178	0	0	0	0	0	0	0	0	0	0	0	62
179	2	57	0	0	0	0	0	0	0	0	0	0
180	0	0	0	0	0	0	0	0	0	60	0	0

OTU Number	7.11.15 Rep1	7.11.15 Rep2	7.11.15 Rep3	8.29.15 Rep1	8.29.15 Rep2	8.29.15 Rep3	2.6.16 Rep1	2.6.16 Rep2	2.6.16 Rep3	12.10.16 Rep1	12.10.16 Rep2	12.10.16 Rep3
181	1	3	0	1	1	1	0	0	0	0	0	0
182	0	0	0	0	0	0	0	0	0	0	60	0
183	0	0	0	24	20	8	0	0	0	0	0	0
184	0	0	0	0	0	0	0	0	0	0	59	0
185	16	5	2	0	3	2	0	0	0	0	0	0
186	19	11	8	1	3	0	0	0	0	0	0	0
187	0	0	0	0	3	0	0	0	0	0	0	0
188	7	5	2	0	0	0	0	0	0	0	0	0
189	0	0	0	0	0	0	0	0	0	0	0	0
190	0	0	0	0	0	0	0	0	0	0	0	0
191	0	0	0	0	0	0	0	0	0	0	0	56
192	0	0	0	0	0	0	0	0	0	0	55	0
193	0	0	0	0	0	0	0	0	0	0	55	0
194	0	0	0	0	0	0	0	0	0	0	0	55
195	0	0	0	0	0	0	0	0	0	0	0	54
196	0	0	0	0	0	0	0	0	0	0	54	0
197	0	0	0	0	0	0	0	0	0	0	0	0
198	12	9	2	0	5	1	0	0	0	0	0	0
199	1	1	0	1	1	0	3	0	0	0	0	0
200	1	2	0	0	0	1	0	0	0	0	0	0
201	0	0	0	0	0	0	0	0	0	0	0	52
202	0	0	0	0	0	0	0	51	0	0	0	0
203	1	1	0	0	0	0	0	0	0	0	0	0
204	0	0	0	0	0	0	0	0	0	50	0	0
205	1	0	0	0	0	0	0	0	0	0	0	0
206	0	0	0	0	0	1	0	0	0	0	0	0
207	7	8	15	2	2	1	0	0	0	0	0	0
208	5	2	0	0	1	2	0	0	0	0	0	0
209	1	0	1	0	0	11	0	0	0	0	0	0
210	5	39	0	0	0	0	0	0	0	0	0	0
211	2	2	1	0	1	2	0	0	0	0	0	0
212	1	0	1	4	4	2	0	0	0	0	0	0
213	0	0	0	0	0	0	0	0	0	0	0	47
214	0	0	0	0	0	0	0	47	0	0	0	0
215	0	0	0	0	0	0	0	0	0	0	0	0
216	0	0	0	0	0	0	0	0	0	0	0	0
217	0	0	0	0	0	0	0	0	0	0	0	0
218	11	30	0	0	0	0	0	0	0	0	0	0
219	0	0	0	0	0	0	0	0	44	0	0	0
220	0	6	0	0	0	1	0	0	0	0	0	0
221	0	0	0	0	0	0	0	0	0	43	0	0
222	0	0	0	0	0	0	0	0	43	0	0	0
223	17	6	2	1	2	0	0	0	0	0	0	0
224	0	0	0	0	0	0	0	0	0	0	0	0
225	0	0	0	0	0	0	0	0	0	0	0	0

OTU Number	7.11.15 Rep1	7.11.15 Rep2	7.11.15 Rep3	8.29.15 Rep1	8.29.15 Rep2	8.29.15 Rep3	2.6.16 Rep1	2.6.16 Rep2	2.6.16 Rep3	12.10.16 Rep1	12.10.16 Rep2	12.10.16 Rep3
226	0	25	0	0	0	0	0	0	0	0	0	0
227	5	8	3	2	0	0	0	0	0	0	0	0
228	0	9	0	0	0	0	0	0	0	0	0	0
229	0	0	0	0	0	0	0	0	0	0	0	39
230	0	0	0	0	0	0	0	0	0	0	39	0
231	0	0	0	0	0	0	0	38	0	0	0	0
232	0	0	0	0	0	0	0	0	0	38	0	0
233	0	0	0	0	0	0	0	38	0	0	0	0
234	0	0	0	0	0	0	0	0	0	0	38	0
235	1	0	2	3	5	5	0	0	0	0	0	0
236	0	0	0	0	0	0	0	0	0	0	38	0
237	0	0	0	0	0	0	0	0	0	0	37	0
238	12	11	2	2	0	2	0	0	0	0	0	0
239	0	0	0	0	0	0	0	0	0	37	0	0
240	0	0	0	0	0	0	0	0	0	0	0	0
241	0	0	0	0	0	0	0	0	0	0	0	0
242	0	0	0	0	0	0	0	0	0	0	0	37
243	0	0	9	15	7	3	0	0	0	0	0	0
244	5	2	0	0	4	6	0	0	0	0	0	0
245	0	0	0	0	0	0	0	0	0	0	36	0
246	0	0	0	1	0	0	0	0	0	0	0	0
247	0	0	0	0	0	0	0	0	0	0	0	0
248	1	1	0	0	1	0	4	0	0	0	0	0
249	0	0	0	0	0	0	0	0	35	0	0	0
250	0	0	1	0	0	0	0	0	0	0	0	0
251	0	0	0	0	0	0	0	0	0	0	34	0
252	0	1	0	0	0	0	0	0	0	0	0	0
253	0	0	0	0	0	0	0	0	0	34	0	0
254	1	26	2	0	0	0	0	0	0	0	0	0
255	0	0	0	0	0	0	0	0	0	0	34	0
256	0	3	0	0	0	0	0	0	0	0	0	0
257	0	0	0	0	0	0	0	0	33	0	0	0
258	1	22	0	0	0	0	0	0	0	0	0	0
259	0	0	0	0	0	0	0	0	0	0	33	0
260	0	0	0	0	0	0	0	0	0	0	0	0
261	5	4	1	0	0	0	0	0	0	0	0	0
262	0	0	0	0	0	0	0	0	0	0	0	32
263	0	0	0	0	0	0	0	0	32	0	0	0
264	5	3	1	1	3	1	0	0	0	0	0	0
265	9	12	1	0	0	0	0	0	0	0	0	0
266	15	9	4	0	0	0	0	0	0	0	0	0
267	0	0	0	0	0	0	0	0	0	0	0	0
268	0	0	0	0	0	0	0	0	0	0	0	32
269	6	3	2	0	1	0	0	0	0	0	0	0
270	0	0	0	0	0	0	0	0	0	0	0	31

OTU Number	7.11.15 Rep1	7.11.15 Rep2	7.11.15 Rep3	8.29.15 Rep1	8.29.15 Rep2	8.29.15 Rep3	2.6.16 Rep1	2.6.16 Rep2	2.6.16 Rep3	12.10.16 Rep1	12.10.16 Rep2	12.10.16 Rep3
271	0	31	0	0	0	0	0	0	0	0	0	0
272	0	0	0	0	0	0	0	31	0	0	0	0
273	0	0	0	0	0	0	0	0	0	0	0	0
274	0	0	0	0	0	0	0	0	31	0	0	0
275	2	7	0	0	3	0	0	0	0	0	0	0
276	0	0	0	0	0	0	0	0	0	0	30	0
277	0	0	0	0	0	0	0	0	0	0	30	0
278	0	0	0	0	0	0	0	0	0	0	30	0
279	0	0	0	0	0	0	0	0	0	0	0	0
280	0	0	0	0	0	0	0	0	0	0	30	0
281	0	0	0	0	0	0	0	0	30	0	0	0
282	0	0	0	0	0	0	0	0	0	0	0	30
283	0	0	0	0	0	0	0	0	0	0	0	30
284	0	0	0	0	0	0	0	0	0	0	0	0
285	0	0	0	0	0	0	0	0	0	0	0	29
286	0	0	0	0	0	0	0	0	0	0	0	29
287	0	0	0	12	2	15	0	0	0	0	0	0
288	0	0	0	0	0	0	0	28	0	0	0	0
289	1	1	0	0	6	0	3	0	0	0	0	0
290	0	0	0	0	0	0	0	0	28	0	0	0
291	0	0	0	0	0	0	0	0	0	0	0	0
292	0	0	0	0	0	0	0	0	0	0	0	28
293	1	1	0	0	0	0	0	0	0	0	0	0
294	0	0	0	0	0	0	0	0	0	28	0	0
295	0	0	0	0	0	0	1	0	0	0	0	0
296	3	1	0	0	0	0	0	0	0	0	0	0
297	0	3	0	0	0	0	0	0	0	0	0	0
298	0	0	0	0	0	0	0	27	0	0	0	0
299	0	0	0	0	0	0	0	0	0	0	27	0
300	0	0	0	0	0	0	0	0	0	0	0	0
301	0	0	0	0	0	0	0	26	0	0	0	0
302	0	0	0	0	0	0	0	0	0	0	0	26
303	0	0	0	0	0	0	0	0	0	0	0	0
304	0	5	0	0	0	0	0	0	0	0	0	0
305	0	0	0	0	0	0	0	0	0	0	0	0
306	0	0	0	0	0	0	0	0	0	0	0	0
307	0	0	0	0	0	0	0	0	26	0	0	0
308	4	11	1	0	0	0	0	0	0	0	0	0
309	1	0	1	7	4	13	0	0	0	0	0	0
310	0	0	0	0	0	0	0	0	26	0	0	0
311	0	0	0	0	0	0	2	0	0	0	0	0
312	0	0	0	0	0	0	0	0	0	0	0	0
313	0	0	0	0	3	3	0	0	0	0	0	0
314	0	0	0	0	0	0	0	0	25	0	0	0
315	0	22	0	0	0	0	0	0	0	0	0	0

OTU Number	7.11.15 Rep1	7.11.15 Rep2	7.11.15 Rep3	8.29.15 Rep1	8.29.15 Rep2	8.29.15 Rep3	2.6.16 Rep1	2.6.16 Rep2	2.6.16 Rep3	12.10.16 Rep1	12.10.16 Rep2	12.10.16 Rep3
316	0	0	0	1	0	0	0	0	0	0	0	0
317	0	0	0	0	0	0	0	0	0	0	25	0
318	0	0	0	0	0	0	0	25	0	0	0	0
319	0	0	0	1	12	2	0	0	0	0	0	0
320	0	0	0	0	0	0	0	0	0	0	0	0
321	0	0	0	0	0	0	0	0	0	0	0	24
322	0	0	0	0	0	0	0	0	0	0	0	24
323	0	0	0	0	0	0	0	0	0	0	0	24
324	0	0	0	0	0	0	0	0	0	0	24	0
325	0	0	0	0	0	0	0	0	0	0	0	0
326	0	0	0	0	0	0	0	0	0	24	0	0
327	0	0	0	0	0	0	0	0	0	0	24	0
328	0	0	0	0	0	0	0	24	0	0	0	0
329	2	1	9	1	2	4	0	0	0	0	0	0
330	0	0	0	0	0	0	0	0	0	0	0	0
331	0	0	0	0	0	0	0	0	0	0	0	0
332	0	0	0	0	0	0	0	0	0	0	0	0
333	0	0	0	0	0	0	0	23	0	0	0	0
334	0	0	0	0	0	0	0	0	0	0	23	0
335	0	0	0	0	0	0	0	0	0	0	0	23
336	1	0	0	4	12	6	0	0	0	0	0	0
337	0	0	0	0	0	0	0	0	0	0	0	23
338	1	0	0	4	3	8	0	0	0	0	0	0
339	0	0	0	0	0	0	0	0	0	0	0	0
340	0	0	0	0	0	0	0	0	0	0	0	0
341	0	0	0	0	0	0	0	0	0	0	0	23
342	0	0	0	0	0	0	0	0	0	0	0	0
343	0	0	0	0	0	0	0	0	0	0	0	0
344	0	0	0	0	0	0	0	23	0	0	0	0
345	2	0	0	0	3	1	0	0	0	0	0	0
346	0	0	0	0	1	0	0	0	0	0	0	0
347	0	0	0	0	0	0	0	0	0	0	22	0
348	0	0	0	0	0	0	0	0	0	0	22	0
349	0	0	0	0	0	0	0	0	0	22	0	0
350	0	0	0	0	0	0	0	0	0	22	0	0
351	0	0	0	0	0	0	0	22	0	0	0	0
352	0	22	0	0	0	0	0	0	0	0	0	0
353	0	0	0	0	0	0	0	0	0	22	0	0
354	0	0	0	0	0	0	0	0	0	0	0	0
355	0	0	0	0	0	0	0	0	0	22	0	0
356	0	0	0	0	0	0	0	0	0	0	0	22
357	0	0	0	0	0	0	0	0	0	0	0	0
358	0	0	0	0	0	0	0	22	0	0	0	0
359	0	0	0	0	0	0	0	22	0	0	0	0
360	0	0	0	0	0	0	0	0	22	0	0	0

OTU Number	7.11.15 Rep1	7.11.15 Rep2	7.11.15 Rep3	8.29.15 Rep1	8.29.15 Rep2	8.29.15 Rep3	2.6.16 Rep1	2.6.16 Rep2	2.6.16 Rep3	12.10.16 Rep1	12.10.16 Rep2	12.10.16 Rep3
361	0	0	0	0	0	0	0	0	0	0	0	0
362	0	0	0	0	0	0	0	0	0	0	0	0
363	0	0	0	0	0	0	0	0	0	0	22	0
364	0	0	0	0	0	0	0	0	0	0	0	0
365	0	0	0	0	0	0	0	0	0	0	22	0
366	0	0	0	0	0	0	0	0	21	0	0	0
367	0	0	0	0	0	0	0	0	0	21	0	0
368	0	0	0	0	0	0	0	0	0	21	0	0
369	0	0	0	0	0	0	0	21	0	0	0	0
370	0	0	0	0	0	0	0	21	0	0	0	0
371	0	0	0	0	0	0	0	0	0	0	0	0
372	0	0	0	0	0	0	0	0	0	21	0	0
373	0	0	0	0	0	0	0	0	0	21	0	0
374	0	0	0	0	0	0	0	0	21	0	0	0
375	0	0	0	0	0	0	0	0	21	0	0	0
376	0	0	0	0	0	0	0	0	0	0	0	0
377	6	5	2	1	2	0	0	0	0	0	0	0
378	0	0	0	0	0	0	0	0	0	0	0	0
379	0	0	0	0	8	0	0	0	0	0	0	0
380	0	0	0	0	0	0	0	0	21	0	0	0
381	0	0	0	0	0	0	0	0	0	0	0	0
382	0	1	0	0	0	0	0	0	0	0	0	0
383	0	0	0	0	0	0	0	0	20	0	0	0
384	1	0	0	0	3	5	0	0	0	0	0	0
385	0	0	0	0	0	0	0	0	0	0	0	0
386	0	0	0	0	0	0	0	0	0	0	20	0
387	0	0	0	0	0	0	0	20	0	0	0	0
388	0	0	0	0	0	0	0	0	0	0	20	0
389	0	0	0	0	1	0	0	0	0	0	0	0
390	0	0	0	0	0	0	0	0	0	0	0	0
391	0	0	0	0	0	0	0	0	0	0	0	0
392	0	0	0	0	0	0	0	0	0	0	20	0
393	0	20	0	0	0	0	0	0	0	0	0	0
394	0	0	0	0	0	0	0	0	0	0	0	0
395	0	0	0	0	0	0	0	0	0	0	0	0
396	0	1	0	0	0	0	0	0	0	0	0	0
397	0	0	0	0	0	0	0	0	0	19	0	0
398	0	0	0	0	0	0	0	0	0	0	0	19
399	0	0	0	0	0	0	0	0	0	0	0	0
400	0	0	0	0	0	0	0	0	0	19	0	0
401	0	2	0	0	0	0	0	0	0	0	0	0
402	0	0	0	0	0	0	0	0	19	0	0	0
403	0	0	0	0	0	0	0	0	19	0	0	0
404	0	0	0	0	0	0	0	19	0	0	0	0
405	2	6	1	0	0	1	0	0	0	0	0	0

OTU Number	7.11.15 Rep1	7.11.15 Rep2	7.11.15 Rep3	8.29.15 Rep1	8.29.15 Rep2	8.29.15 Rep3	2.6.16 Rep1	2.6.16 Rep2	2.6.16 Rep3	12.10.16 Rep1	12.10.16 Rep2	12.10.16 Rep3
406	0	0	0	0	0	0	0	0	19	0	0	0
407	0	0	0	0	0	0	0	0	19	0	0	0
408	0	0	0	0	0	0	0	0	0	19	0	0
409	0	0	0	0	0	0	0	0	0	0	0	0
410	2	3	1	4	3	1	0	0	0	0	0	0
411	0	0	0	0	0	0	0	19	0	0	0	0
412	0	0	0	0	0	0	0	0	0	0	0	0
413	0	0	0	0	0	0	0	0	0	0	0	0
414	5	8	0	0	0	1	0	0	0	0	0	0
415	0	0	0	0	0	0	0	0	0	0	0	0
416	0	0	0	0	0	0	0	19	0	0	0	0
417	0	1	0	0	2	0	0	0	0	0	0	0
418	0	0	0	0	0	0	0	0	19	0	0	0
419	0	0	0	0	0	0	0	0	0	0	0	0
420	0	0	0	0	0	0	0	0	0	0	18	0
421	0	0	0	0	0	0	0	0	0	0	0	0
422	0	0	0	0	0	0	0	18	0	0	0	0
423	1	15	0	0	0	0	0	0	0	0	0	0
424	0	0	0	0	0	0	0	18	0	0	0	0
425	0	0	0	0	0	0	0	0	0	0	0	0
426	0	0	0	0	0	0	0	0	0	0	0	0
427	0	0	0	0	0	0	0	0	0	0	18	0
428	0	1	1	2	0	0	0	0	0	0	0	0
429	0	0	0	0	0	0	0	18	0	0	0	0
430	0	0	0	0	0	0	0	0	0	0	0	0
431	0	0	0	0	0	0	0	0	0	18	0	0
432	0	0	0	0	0	0	0	0	18	0	0	0
433	0	0	0	0	0	0	0	0	0	0	0	0
434	0	0	0	0	0	0	0	0	0	0	0	0
435	0	0	0	0	0	0	0	0	0	0	0	0
436	0	0	0	0	0	0	0	0	18	0	0	0
437	0	0	0	0	0	0	0	0	0	0	0	0
438	0	0	0	0	0	0	0	18	0	0	0	0
439	0	6	0	0	0	0	0	0	0	0	0	0
440	0	0	0	0	0	0	0	0	0	0	0	18
441	0	0	0	0	0	0	0	0	0	0	0	18
442	0	0	0	0	0	0	0	0	18	0	0	0
443	1	0	0	0	0	0	0	0	0	0	0	0
444	0	0	0	0	0	0	0	0	18	0	0	0
445	0	0	0	0	0	0	0	0	0	0	0	18
446	0	0	0	0	0	0	0	18	0	0	0	0
447	0	0	0	0	0	0	0	0	0	18	0	0
448	0	0	0	0	0	0	0	0	0	0	0	0
449	0	0	0	0	0	0	0	0	0	18	0	0
450	0	0	0	0	0	0	0	17	0	0	0	0

OTU Number	7.11.15 Rep1	7.11.15 Rep2	7.11.15 Rep3	8.29.15 Rep1	8.29.15 Rep2	8.29.15 Rep3	2.6.16 Rep1	2.6.16 Rep2	2.6.16 Rep3	12.10.16 Rep1	12.10.16 Rep2	12.10.16 Rep3
451	0	0	0	0	0	0	0	0	0	17	0	0
452	0	0	0	0	0	0	0	0	17	0	0	0
453	0	0	0	0	0	0	0	0	0	0	17	0
454	0	0	0	0	0	0	0	0	17	0	0	0
455	0	0	0	0	4	1	0	0	0	0	0	0
456	0	0	0	0	0	0	0	0	0	0	0	0
457	0	0	0	0	0	0	0	0	0	0	0	17
458	0	0	0	0	0	0	0	0	0	0	0	0
459	0	0	0	0	0	0	0	0	0	0	0	17
460	0	0	0	0	0	0	0	0	0	0	0	0
461	0	0	0	0	0	0	0	0	0	0	17	0
462	0	0	0	0	0	0	0	0	0	0	0	0
463	0	0	0	0	0	0	0	0	0	17	0	0
464	0	0	0	0	0	0	0	0	0	0	0	0
465	0	2	0	0	4	2	0	0	0	0	0	0
466	0	0	0	0	0	0	0	0	0	0	0	0
467	0	0	0	0	0	0	0	17	0	0	0	0
468	0	0	0	0	0	0	0	0	0	0	0	17
469	1	0	4	0	3	8	0	0	0	0	0	0
470	0	0	0	0	0	0	0	0	17	0	0	0
471	0	0	0	0	0	0	0	0	17	0	0	0
472	0	0	0	1	0	0	1	0	0	0	0	0
473	0	0	0	0	0	0	0	0	0	0	0	0
474	0	0	0	0	0	0	0	0	0	0	0	0
475	0	0	0	0	0	0	0	0	17	0	0	0
476	0	0	0	0	0	0	0	17	0	0	0	0
477	0	0	0	0	0	0	0	0	0	0	0	0
478	0	0	0	0	0	0	0	0	0	0	0	0
479	0	0	0	0	0	0	0	0	0	16	0	0
480	0	0	0	0	0	0	0	0	0	0	0	16
481	0	0	0	0	0	0	0	0	0	0	0	0
482	0	0	0	0	2	0	0	0	0	0	0	0
483	0	0	0	0	0	0	0	0	16	0	0	0
484	0	0	0	0	0	0	0	0	0	0	0	0
485	0	0	0	0	0	0	0	0	0	16	0	0
486	0	0	0	0	0	0	0	0	16	0	0	0
487	0	0	0	0	0	0	0	0	0	16	0	0
488	0	0	0	0	0	0	0	0	0	16	0	0
489	0	0	0	0	0	0	0	0	0	0	0	16
490	3	2	2	0	0	1	0	0	0	0	0	0
491	0	0	0	0	0	0	0	0	16	0	0	0
492	0	0	0	0	0	0	0	16	0	0	0	0
493	0	0	0	0	0	0	0	0	0	16	0	0
494	0	0	0	0	0	0	0	0	0	0	0	0
495	0	0	0	0	0	0	0	0	0	0	16	0

OTU Number	7.11.15 Rep1	7.11.15 Rep2	7.11.15 Rep3	8.29.15 Rep1	8.29.15 Rep2	8.29.15 Rep3	2.6.16 Rep1	2.6.16 Rep2	2.6.16 Rep3	12.10.16 Rep1	12.10.16 Rep2	12.10.16 Rep3
496	0	0	0	0	0	0	0	16	0	0	0	0
497	0	0	0	0	0	0	0	0	0	0	0	0
498	0	0	0	0	2	1	0	0	0	0	0	0
499	0	0	0	0	0	0	0	16	0	0	0	0
500	0	0	0	0	0	0	0	0	0	0	16	0
501	0	0	0	0	0	0	0	0	0	16	0	0
502	0	0	0	0	0	0	0	0	16	0	0	0
503	0	0	0	0	0	0	0	0	0	0	0	16
504	0	0	0	0	0	0	0	0	0	16	0	0
505	9	4	0	0	0	0	0	0	0	0	0	0
506	0	0	0	0	0	0	0	0	0	0	0	0
507	0	0	0	0	0	0	0	0	0	0	0	16
508	0	0	0	0	0	0	0	0	0	0	0	0
509	1	0	0	0	0	0	0	0	0	0	0	0
510	0	0	0	0	0	0	0	0	0	15	0	0
511	0	0	0	0	0	0	0	0	0	0	0	0
512	0	0	0	3	3	4	0	0	0	0	0	0
513	0	0	0	0	0	0	0	0	0	0	0	0
514	0	0	0	0	0	1	0	0	0	0	0	0
515	0	0	0	0	0	0	0	0	0	0	0	0
516	0	0	0	0	0	0	0	0	15	0	0	0
517	0	0	0	0	0	0	0	0	0	0	0	0
518	0	15	0	0	0	0	0	0	0	0	0	0
519	0	0	0	0	0	0	0	0	0	0	0	15
520	0	0	0	0	0	0	0	0	0	15	0	0
521	2	2	2	0	0	0	0	0	0	0	0	0
522	0	0	0	0	0	0	0	15	0	0	0	0
523	0	0	0	0	0	0	0	0	0	0	0	0
524	0	0	0	0	0	0	0	15	0	0	0	0
525	0	0	0	0	0	0	0	0	0	0	0	0
526	0	0	0	0	0	0	0	0	0	15	0	0
527	0	0	0	0	0	0	0	0	0	0	0	0
528	0	0	0	0	0	0	0	15	0	0	0	0
529	0	0	0	0	0	0	0	0	0	0	0	0
530	0	0	0	0	0	0	0	15	0	0	0	0
531	0	0	0	0	0	0	0	0	15	0	0	0
532	0	0	0	0	0	0	0	0	0	15	0	0
533	0	0	0	0	0	0	0	0	15	0	0	0
534	0	0	0	0	0	0	0	0	0	0	0	0
535	0	0	0	0	0	0	0	0	15	0	0	0
536	0	0	0	0	0	0	0	0	0	0	0	15
537	0	0	0	0	0	0	0	0	0	0	0	0
538	0	0	0	0	0	0	0	0	0	0	15	0
539	0	0	0	0	0	0	0	0	0	15	0	0
540	0	0	0	0	0	0	0	0	0	14	0	0

OTU Number	7.11.15 Rep1	7.11.15 Rep2	7.11.15 Rep3	8.29.15 Rep1	8.29.15 Rep2	8.29.15 Rep3	2.6.16 Rep1	2.6.16 Rep2	2.6.16 Rep3	12.10.16 Rep1	12.10.16 Rep2	12.10.16 Rep3
541	0	0	0	0	0	0	0	14	0	0	0	0
542	0	0	0	0	0	0	0	0	0	14	0	0
543	0	0	0	0	0	0	0	0	0	0	0	0
544	0	0	0	0	0	0	4	0	0	0	0	0
545	0	0	0	0	0	0	0	0	14	0	0	0
546	0	0	0	0	0	0	0	0	0	0	0	0
547	0	0	0	0	0	0	0	0	0	0	0	0
548	0	0	0	0	0	0	0	0	0	0	0	0
549	0	0	0	0	0	0	0	0	0	0	14	0
550	0	0	0	0	0	0	0	0	14	0	0	0
551	2	0	0	0	0	0	0	0	0	0	0	0
552	0	0	0	0	0	0	0	0	0	14	0	0
553	0	0	0	0	0	0	0	0	0	0	14	0
554	0	0	0	0	0	0	0	0	0	14	0	0
555	0	0	0	0	0	0	0	0	0	0	0	14
556	3	0	3	2	1	5	0	0	0	0	0	0
557	0	0	0	0	0	0	0	0	0	0	0	14
558	0	0	3	1	3	7	0	0	0	0	0	0
559	0	0	0	0	0	0	0	0	0	0	14	0
560	0	0	0	0	0	0	0	0	0	14	0	0
561	0	0	0	0	0	0	0	0	0	0	0	14
562	0	0	0	0	0	0	0	0	0	0	0	0
563	0	0	0	0	0	0	0	0	0	14	0	0
564	0	0	0	0	0	0	0	0	0	0	0	0
565	0	0	0	0	0	0	0	0	0	0	0	0
566	5	0	0	0	0	0	0	0	0	0	0	0
567	0	0	0	0	0	0	0	0	0	14	0	0
568	1	2	0	0	1	1	0	0	0	0	0	0
569	0	0	0	0	0	0	0	0	0	0	0	0
570	0	0	0	0	0	0	0	0	14	0	0	0
571	0	0	0	0	0	0	0	0	0	0	0	0
572	0	1	0	0	0	0	0	0	0	0	0	0
573	0	0	0	0	0	0	0	0	0	14	0	0
574	4	2	0	0	0	0	0	0	0	0	0	0
575	0	0	0	0	0	0	0	0	0	14	0	0
576	0	0	0	0	0	0	0	0	0	14	0	0
577	0	0	0	0	0	0	0	0	0	0	0	14
578	0	0	0	1	1	0	0	0	0	0	0	0
579	0	0	0	0	0	0	0	0	0	14	0	0
580	0	0	0	0	0	0	0	0	0	0	0	0
581	0	1	0	0	0	0	1	0	0	0	0	0
582	0	0	0	0	0	0	0	0	0	14	0	0
583	0	0	0	0	0	0	0	0	0	14	0	0
584	0	0	0	0	0	0	0	14	0	0	0	0
585	0	0	0	0	0	0	0	0	0	0	0	0

OTU Number	7.11.15 Rep1	7.11.15 Rep2	7.11.15 Rep3	8.29.15 Rep1	8.29.15 Rep2	8.29.15 Rep3	2.6.16 Rep1	2.6.16 Rep2	2.6.16 Rep3	12.10.16 Rep1	12.10.16 Rep2	12.10.16 Rep3
586	0	0	0	0	0	0	0	0	0	14	0	0
587	0	0	0	0	0	0	0	0	0	13	0	0
588	0	0	0	0	0	0	0	0	0	0	13	0
589	0	0	0	0	0	0	0	0	0	0	13	0
590	0	0	0	0	0	0	0	0	0	13	0	0
591	0	0	0	0	0	0	0	0	0	13	0	0
592	0	0	0	0	0	0	0	0	0	0	0	0
593	0	0	0	0	0	0	0	0	0	0	0	0
594	0	0	0	0	0	0	0	0	0	0	0	13
595	0	0	0	0	0	0	0	0	0	0	0	0
596	0	0	0	0	0	0	0	0	0	0	0	13
597	0	1	1	0	1	0	0	0	0	0	0	0
598	0	0	0	0	0	0	0	0	0	0	0	0
599	0	0	0	0	0	0	0	13	0	0	0	0
600	0	0	0	0	0	0	0	0	0	0	13	0
601	0	0	0	0	0	0	0	0	0	0	0	0
602	0	0	0	0	0	0	0	0	0	0	0	0
603	0	0	0	0	0	0	0	13	0	0	0	0
604	0	0	0	0	0	0	0	13	0	0	0	0
605	0	0	0	0	0	0	0	0	0	0	0	0
606	0	0	0	0	0	0	0	13	0	0	0	0
607	0	0	0	0	0	0	0	0	0	0	0	0
608	4	1	2	0	0	0	0	0	0	0	0	0
609	0	0	0	0	0	0	0	0	0	0	0	0
610	0	0	0	0	0	0	0	0	0	0	0	0
611	0	0	0	0	0	0	0	0	0	13	0	0
612	0	0	0	0	0	0	0	0	0	13	0	0
613	0	0	0	0	0	0	0	0	0	0	0	0
614	0	0	0	0	0	0	0	0	13	0	0	0
615	0	0	0	0	0	0	0	0	0	13	0	0
616	0	0	0	0	0	0	0	0	0	13	0	0
617	0	0	0	0	0	0	0	13	0	0	0	0
618	0	0	0	0	0	0	0	0	0	13	0	0
619	0	0	0	0	0	0	0	13	0	0	0	0
620	0	0	0	0	0	0	0	0	0	13	0	0
621	0	0	0	0	0	0	0	13	0	0	0	0
622	0	0	0	0	0	0	0	0	0	13	0	0
623	0	0	0	0	0	0	0	0	0	0	0	0
624	0	0	0	0	0	0	0	0	0	13	0	0
625	0	0	0	0	0	0	0	0	0	0	13	0
626	0	0	0	0	0	0	0	0	0	0	0	0
627	0	0	0	0	0	0	0	0	13	0	0	0
628	0	0	0	0	0	0	0	13	0	0	0	0
629	0	0	0	0	0	0	0	13	0	0	0	0
630	0	0	0	0	0	0	0	0	0	0	13	0

OTU Number	7.11.15 Rep1	7.11.15 Rep2	7.11.15 Rep3	8.29.15 Rep1	8.29.15 Rep2	8.29.15 Rep3	2.6.16 Rep1	2.6.16 Rep2	2.6.16 Rep3	12.10.16 Rep1	12.10.16 Rep2	12.10.16 Rep3
631	0	0	0	0	0	0	0	0	0	0	0	0
632	0	0	0	0	0	0	0	0	0	13	0	0
633	0	0	0	0	0	0	0	0	0	0	13	0
634	0	0	0	0	0	0	0	0	13	0	0	0
635	0	0	0	0	0	0	0	13	0	0	0	0
636	0	0	0	0	0	0	0	0	13	0	0	0
637	0	0	0	0	0	0	0	0	0	13	0	0
638	0	0	0	0	0	0	0	0	13	0	0	0
639	0	0	0	0	0	0	0	0	0	0	0	0
640	0	0	0	0	0	0	0	0	13	0	0	0
641	0	0	0	0	0	0	0	0	0	13	0	0
642	0	0	0	0	0	0	0	0	0	13	0	0
643	0	0	0	0	0	0	0	0	12	0	0	0
644	0	0	0	0	0	0	0	12	0	0	0	0
645	0	0	0	0	0	0	0	0	12	0	0	0
646	0	0	0	0	0	0	0	0	0	12	0	0
647	0	0	0	0	0	0	0	0	0	0	0	0
648	0	0	0	0	0	0	0	12	0	0	0	0
649	0	0	0	0	0	0	0	0	0	0	12	0
650	0	0	0	0	0	0	0	0	12	0	0	0
651	0	0	0	0	0	0	0	0	12	0	0	0
652	0	0	0	0	0	0	0	0	0	0	12	0
653	2	1	2	0	1	0	0	0	0	0	0	0
654	5	0	7	0	0	0	0	0	0	0	0	0
655	0	0	0	0	0	0	0	0	0	0	0	0
656	0	0	0	0	0	0	0	12	0	0	0	0
657	0	3	0	0	0	0	0	0	0	0	0	0
658	0	0	0	0	0	0	0	0	0	0	0	0
659	0	0	0	0	0	0	0	12	0	0	0	0
660	0	0	0	0	0	0	0	0	12	0	0	0
661	0	0	0	0	0	0	0	0	0	0	12	0
662	0	0	0	0	0	0	0	0	0	0	0	0
663	0	0	0	0	0	0	0	0	0	0	0	12
664	0	0	0	0	0	0	0	0	0	0	0	12
665	1	2	0	0	0	0	0	0	0	0	0	0
666	0	0	0	0	0	0	0	0	0	0	0	0
667	0	0	0	0	0	0	0	0	0	0	0	12
668	0	0	0	0	0	0	0	0	12	0	0	0
669	0	0	0	0	0	0	0	0	0	0	0	0
670	0	0	0	0	0	0	0	0	0	0	0	12
671	0	0	0	0	0	0	0	0	0	0	12	0
672	0	0	0	0	0	0	0	0	0	0	0	0
673	0	0	0	0	0	0	0	0	0	0	0	0
674	0	0	0	0	0	0	0	0	0	0	0	12
675	0	0	0	0	0	0	0	0	0	0	0	0

OTU Number	7.11.15 Rep1	7.11.15 Rep2	7.11.15 Rep3	8.29.15 Rep1	8.29.15 Rep2	8.29.15 Rep3	2.6.16 Rep1	2.6.16 Rep2	2.6.16 Rep3	12.10.16 Rep1	12.10.16 Rep2	12.10.16 Rep3
676	0	0	0	0	0	0	0	0	0	0	0	12
677	0	0	0	0	0	0	0	0	12	0	0	0
678	0	0	0	0	0	0	0	0	0	0	12	0
679	0	0	0	0	0	0	0	0	0	0	0	12
680	0	0	0	0	0	0	0	0	12	0	0	0
681	0	0	0	0	0	0	0	0	12	0	0	0
682	0	0	0	0	0	0	0	0	0	0	0	12
683	0	0	0	0	0	0	0	12	0	0	0	0
684	0	0	0	0	0	0	0	0	0	12	0	0
685	0	0	0	0	0	0	0	0	0	0	0	0
686	0	0	0	0	0	0	0	0	12	0	0	0
687	6	0	3	0	1	0	0	0	0	0	0	0
688	0	0	0	0	0	0	0	0	0	0	0	0
689	1	0	0	0	2	1	0	0	0	0	0	0
690	0	0	0	0	0	0	0	0	12	0	0	0
691	0	0	0	0	0	0	0	0	0	0	0	0
692	0	0	0	0	0	0	0	12	0	0	0	0
693	0	0	0	0	0	0	0	0	0	0	0	0
694	0	0	0	0	0	0	0	0	0	0	0	12
695	0	0	0	0	0	0	0	0	12	0	0	0
696	0	0	0	0	0	0	0	12	0	0	0	0
697	0	0	0	0	0	0	0	0	0	0	0	0
698	0	0	0	0	0	0	0	0	0	0	0	0
699	0	0	5	0	0	1	0	0	0	0	0	0
700	0	0	0	0	0	0	0	0	0	0	0	0
701	1	0	5	1	0	0	0	0	0	0	0	0
702	0	0	0	0	0	0	0	12	0	0	0	0
703	0	0	0	2	6	0	0	0	0	0	0	0
704	0	0	0	0	0	0	0	0	12	0	0	0
705	0	0	0	0	0	0	0	0	0	12	0	0
706	0	0	0	0	0	0	0	0	0	0	12	0
707	0	0	0	0	0	0	0	0	12	0	0	0
708	0	0	0	0	0	0	0	12	0	0	0	0
709	0	0	0	0	0	0	0	12	0	0	0	0
710	0	0	0	0	0	0	0	12	0	0	0	0
711	0	0	0	0	0	0	0	0	0	0	0	0
712	0	0	0	0	0	0	0	12	0	0	0	0
713	0	0	0	0	0	0	0	12	0	0	0	0
714	0	0	0	0	0	0	0	0	0	12	0	0
715	0	0	0	0	0	0	0	0	0	0	0	0
716	0	0	0	0	0	0	0	0	0	0	12	0
717	0	0	0	0	0	0	0	0	0	0	0	0
718	0	0	0	0	0	0	0	0	0	0	0	0
719	4	0	0	0	1	1	0	0	0	0	0	0
720	0	0	0	0	0	0	6	0	0	0	0	0

OTU Number	7.11.15 Rep1	7.11.15 Rep2	7.11.15 Rep3	8.29.15 Rep1	8.29.15 Rep2	8.29.15 Rep3	2.6.16 Rep1	2.6.16 Rep2	2.6.16 Rep3	12.10.16 Rep1	12.10.16 Rep2	12.10.16 Rep3
721	0	0	0	0	0	0	0	12	0	0	0	0
722	0	1	0	1	1	0	0	0	0	0	0	0
723	0	0	0	0	0	0	0	0	0	0	11	0
724	0	0	0	0	0	0	0	0	0	0	0	0
725	0	1	0	0	0	0	0	0	0	0	0	0
726	0	0	0	0	0	0	0	0	0	0	11	0
727	0	0	0	0	0	0	0	0	0	0	0	0
728	0	0	0	0	0	0	0	11	0	0	0	0
729	0	0	0	0	0	0	0	0	0	0	0	0
730	0	0	0	0	0	0	0	0	0	0	0	0
731	0	0	0	0	0	0	0	0	11	0	0	0
732	0	0	0	0	0	0	0	0	11	0	0	0
733	0	0	0	0	0	0	0	0	0	0	0	0
734	0	0	0	0	0	0	0	0	0	0	0	0
735	0	0	0	0	0	0	0	11	0	0	0	0
736	0	0	0	0	0	0	0	0	0	11	0	0
737	0	0	0	0	0	0	0	0	0	0	0	0
738	0	3	1	0	0	1	0	0	0	0	0	0
739	1	0	0	0	0	0	0	0	0	0	0	0
740	0	0	0	0	0	0	0	11	0	0	0	0
741	0	0	0	0	0	0	0	0	0	11	0	0
742	0	0	0	0	0	0	0	0	0	0	11	0
743	0	0	0	0	0	0	0	0	0	11	0	0
744	0	0	0	0	0	0	0	11	0	0	0	0
745	0	0	0	0	0	0	0	0	0	0	0	0
746	0	0	0	0	0	0	0	0	11	0	0	0
747	0	0	0	0	0	0	0	11	0	0	0	0
748	0	0	0	0	0	0	0	0	0	0	11	0
749	0	0	0	0	0	0	0	0	0	11	0	0
750	0	0	0	0	0	0	0	0	0	0	0	11
751	0	0	0	0	0	0	0	0	0	0	0	0
752	0	0	0	0	0	0	0	0	11	0	0	0
753	0	0	0	0	0	0	0	0	0	0	0	0
754	0	0	0	0	0	0	0	0	0	0	0	0
755	0	0	0	0	0	0	0	0	0	0	0	0
756	0	0	0	0	0	0	0	0	0	0	0	0
757	0	0	0	0	0	0	0	0	0	0	0	0
758	0	0	0	0	0	0	0	0	0	11	0	0
759	0	0	0	0	0	0	0	0	0	0	0	11
760	0	0	0	0	0	0	0	0	0	11	0	0
761	0	0	0	0	0	0	0	11	0	0	0	0
762	0	0	0	0	0	0	0	0	11	0	0	0
763	0	0	0	0	0	0	0	0	11	0	0	0
764	0	0	0	0	0	0	0	0	0	0	0	0
765	0	0	0	0	0	0	0	0	0	0	11	0

OTU Number	7.11.15 Rep1	7.11.15 Rep2	7.11.15 Rep3	8.29.15 Rep1	8.29.15 Rep2	8.29.15 Rep3	2.6.16 Rep1	2.6.16 Rep2	2.6.16 Rep3	12.10.16 Rep1	12.10.16 Rep2	12.10.16 Rep3
766	0	0	0	0	0	0	0	11	0	0	0	0
767	0	0	0	0	0	0	0	11	0	0	0	0
768	0	0	0	0	0	0	0	0	0	0	0	0
769	0	0	0	0	0	0	0	0	0	0	0	0
770	2	0	0	0	0	0	0	0	0	0	0	0
771	0	0	0	0	2	1	0	0	0	0	0	0
772	0	0	0	0	0	0	0	0	11	0	0	0
773	0	0	0	0	0	0	0	0	0	0	0	11
774	0	0	0	0	0	0	0	0	0	0	0	0
775	0	0	0	0	0	0	0	0	0	0	11	0
776	0	0	0	0	0	0	0	0	0	0	0	0
777	0	0	0	0	0	0	0	0	0	0	0	0
778	0	0	0	0	0	0	0	0	0	11	0	0
779	0	2	0	0	0	9	0	0	0	0	0	0
780	0	0	0	0	0	0	0	0	0	0	0	0
781	0	0	0	0	0	0	0	0	0	0	0	0
782	1	1	3	0	0	2	0	0	0	0	0	0
783	0	0	0	0	0	0	0	0	0	0	0	0
784	0	0	0	0	0	0	0	0	0	0	11	0
785	0	0	0	0	0	0	0	0	0	0	11	0
786	0	0	0	0	0	0	0	0	0	11	0	0
787	0	0	0	0	0	0	0	0	0	11	0	0
788	0	0	0	0	0	0	0	0	0	0	0	0
789	0	0	0	0	0	0	0	0	0	0	0	0
790	0	0	0	0	0	0	0	0	11	0	0	0
791	1	2	0	0	0	0	0	0	0	0	0	0
792	0	0	0	0	0	0	0	0	0	0	0	0
793	0	0	0	0	0	0	0	0	0	0	0	0
794	0	0	0	0	0	0	0	11	0	0	0	0
795	0	0	0	0	0	0	0	0	0	0	0	0
796	0	0	0	0	0	0	0	11	0	0	0	0
797	0	0	0	0	0	0	0	0	11	0	0	0
798	0	0	0	0	0	0	0	0	11	0	0	0
799	0	0	0	0	0	0	0	0	11	0	0	0
800	2	0	0	0	0	0	3	0	0	0	0	0
801	0	0	0	0	0	0	0	0	0	0	11	0
802	0	0	0	0	0	0	0	0	11	0	0	0
803	0	0	0	0	0	0	0	11	0	0	0	0
804	0	0	0	0	0	0	0	0	0	0	0	0
805	0	0	0	0	0	0	0	0	0	11	0	0
806	0	0	0	0	0	0	0	11	0	0	0	0
807	0	0	0	0	0	0	0	0	0	0	0	0
808	0	0	0	0	0	0	0	0	11	0	0	0
809	0	0	0	0	0	0	0	0	0	0	0	0
810	0	0	0	0	0	0	0	0	0	11	0	0

OTU Number	7.11.15 Rep1	7.11.15 Rep2	7.11.15 Rep3	8.29.15 Rep1	8.29.15 Rep2	8.29.15 Rep3	2.6.16 Rep1	2.6.16 Rep2	2.6.16 Rep3	12.10.16 Rep1	12.10.16 Rep2	12.10.16 Rep3
811	0	0	0	0	0	0	0	0	0	0	0	0
812	0	0	0	0	0	0	0	0	11	0	0	0
813	0	0	0	0	0	0	0	0	0	0	0	0
814	0	0	0	0	0	0	0	0	0	11	0	0
815	0	0	0	0	0	0	0	11	0	0	0	0
816	0	2	0	0	0	0	0	0	0	0	0	0
817	0	0	0	0	0	0	0	0	0	0	0	11
818	0	0	0	0	0	0	0	11	0	0	0	0
819	0	0	0	0	0	0	0	11	0	0	0	0
820	0	0	0	0	0	0	0	0	11	0	0	0
821	0	0	0	0	0	0	0	0	0	0	0	11
822	0	0	0	0	0	0	0	0	0	0	0	11
823	0	0	0	0	0	0	0	0	0	0	0	0
824	0	0	0	0	0	0	0	11	0	0	0	0
825	0	0	0	0	0	0	0	0	0	11	0	0
826	0	0	0	0	0	0	0	0	0	0	0	0
827	0	0	0	0	0	0	0	10	0	0	0	0
828	0	0	0	0	0	0	0	10	0	0	0	0
829	0	0	0	0	0	0	0	10	0	0	0	0
830	0	0	0	0	0	0	0	0	0	0	0	10
831	0	0	0	0	0	0	0	0	0	0	10	0
832	0	0	0	0	0	0	0	0	0	0	0	0
833	0	0	0	0	0	0	0	0	0	0	10	0
834	0	0	0	0	0	0	0	0	0	0	0	0
835	0	0	0	0	0	0	0	0	0	0	0	10
836	0	0	0	0	0	0	0	0	0	0	10	0
837	0	0	0	0	0	0	0	0	0	0	0	10
838	0	0	0	0	0	0	0	0	0	0	0	0
839	0	0	0	0	0	0	0	0	10	0	0	0
840	0	0	0	0	0	0	0	0	10	0	0	0
841	0	0	0	0	0	0	0	0	10	0	0	0
842	0	0	0	0	0	0	0	0	10	0	0	0
843	0	0	0	0	0	0	0	0	0	0	0	0
844	0	0	0	0	0	0	0	0	10	0	0	0
845	0	0	0	0	0	0	0	0	0	0	0	0
846	0	0	0	0	0	0	0	0	10	0	0	0
847	1	0	0	0	0	0	7	0	0	0	0	0
848	0	0	0	0	0	0	0	0	0	0	10	0
849	0	0	0	3	0	1	0	0	0	0	0	0
850	1	0	6	1	0	2	0	0	0	0	0	0
851	7	0	3	0	0	0	0	0	0	0	0	0
852	0	0	0	0	0	0	0	0	10	0	0	0
853	0	0	0	0	0	0	0	0	10	0	0	0
854	0	0	0	0	0	0	0	0	0	0	10	0
855	0	0	0	0	0	0	0	0	10	0	0	0

OTU Number	7.11.15 Rep1	7.11.15 Rep2	7.11.15 Rep3	8.29.15 Rep1	8.29.15 Rep2	8.29.15 Rep3	2.6.16 Rep1	2.6.16 Rep2	2.6.16 Rep3	12.10.16 Rep1	12.10.16 Rep2	12.10.16 Rep3
856	0	0	0	0	0	0	0	0	10	0	0	0
857	0	0	0	0	0	0	0	0	10	0	0	0
858	0	0	0	0	0	0	0	0	0	0	0	0
859	0	0	0	0	0	0	0	0	0	0	0	0
860	0	0	0	0	0	0	0	0	0	0	10	0
861	0	0	0	0	0	0	0	0	0	0	10	0
862	0	0	0	0	0	0	0	0	10	0	0	0
863	0	0	0	0	0	0	0	0	10	0	0	0
864	0	0	0	0	0	0	0	0	10	0	0	0
865	0	0	0	0	0	0	0	0	0	0	0	10
866	0	0	0	0	0	0	0	0	10	0	0	0
867	0	0	0	0	0	0	0	0	10	0	0	0
868	0	0	0	0	0	0	0	0	10	0	0	0
869	0	0	0	0	0	0	0	0	0	0	10	0
870	0	0	0	0	0	0	0	0	10	0	0	0
871	0	0	0	0	0	0	0	0	0	0	0	0
872	0	0	0	0	0	0	0	0	10	0	0	0
873	0	0	0	0	0	0	0	0	0	0	0	0
874	0	0	0	0	0	0	0	0	10	0	0	0
875	0	0	0	0	0	0	0	0	10	0	0	0
876	0	0	0	0	0	0	0	0	0	0	0	0
877	1	0	0	0	0	0	0	0	0	0	0	0
878	0	0	0	0	0	0	0	0	0	10	0	0
879	2	0	0	0	0	0	0	0	0	0	0	0
880	0	0	0	0	0	0	0	10	0	0	0	0
881	0	0	0	0	0	0	0	0	0	10	0	0
882	0	0	0	0	0	0	0	0	0	10	0	0
883	0	0	0	0	0	0	0	10	0	0	0	0
884	0	0	0	0	0	0	0	0	0	0	0	0
885	0	0	0	0	0	0	0	10	0	0	0	0
886	0	0	0	0	0	0	0	0	0	10	0	0
887	0	0	0	0	0	0	0	0	0	10	0	0
888	0	0	0	0	0	0	0	0	0	0	0	10
889	0	0	0	0	0	0	0	0	0	10	0	0
890	0	0	0	0	0	0	0	0	0	10	0	0
891	0	0	0	0	0	0	0	10	0	0	0	0
892	0	0	0	0	0	0	0	0	0	10	0	0
893	0	0	0	0	0	0	0	0	0	0	10	0
894	0	0	0	0	0	0	0	0	0	10	0	0
895	0	0	0	0	0	1	0	0	0	0	0	0
896	0	0	0	0	0	0	0	0	0	10	0	0
897	0	0	0	0	0	0	0	0	0	10	0	0
898	0	0	0	0	0	0	0	0	0	0	0	0
899	0	0	0	0	0	0	0	0	0	10	0	0
900	0	0	0	0	0	0	0	10	0	0	0	0

OTU Number	7.11.15 Rep1	7.11.15 Rep2	7.11.15 Rep3	8.29.15 Rep1	8.29.15 Rep2	8.29.15 Rep3	2.6.16 Rep1	2.6.16 Rep2	2.6.16 Rep3	12.10.16 Rep1	12.10.16 Rep2	12.10.16 Rep3
901	0	0	0	0	0	0	0	10	0	0	0	0
902	1	0	0	0	2	1	0	0	0	0	0	0
903	0	0	0	0	0	0	0	0	0	10	0	0
904	1	0	0	2	1	4	0	0	0	0	0	0
905	0	0	0	0	0	0	0	0	10	0	0	0
906	1	1	0	2	1	1	0	0	0	0	0	0
907	0	0	0	0	0	0	0	10	0	0	0	0
908	0	0	0	0	0	0	0	10	0	0	0	0
909	0	0	0	0	0	0	0	0	0	0	10	0
910	0	0	0	0	0	0	0	0	0	10	0	0
911	0	0	0	0	0	0	0	0	0	10	0	0
912	0	0	0	0	0	0	0	10	0	0	0	0
913	0	0	0	0	0	0	0	0	0	10	0	0
914	0	1	0	0	0	0	0	0	0	0	0	0
915	0	0	0	0	0	0	0	0	0	10	0	0
916	0	0	0	0	0	0	0	10	0	0	0	0
917	0	0	0	0	0	0	0	0	0	10	0	0
918	0	0	0	0	0	0	0	10	0	0	0	0
919	0	0	0	0	0	0	0	10	0	0	0	0
920	0	0	0	0	7	0	0	0	0	0	0	0
921	0	0	0	0	0	0	0	0	0	10	0	0
922	0	0	0	0	0	0	0	0	0	10	0	0
923	0	4	0	0	0	0	0	0	0	0	0	0
924	0	0	0	0	0	0	0	0	0	0	0	0
925	0	0	0	0	0	0	0	10	0	0	0	0
926	0	0	0	0	0	0	0	0	9	0	0	0
927	0	0	0	0	0	0	0	0	0	9	0	0
928	0	0	0	0	0	0	0	0	0	0	0	9
929	0	0	0	0	0	0	0	0	0	9	0	0
930	0	0	0	0	0	0	0	9	0	0	0	0
931	0	0	0	0	0	0	0	0	9	0	0	0
932	0	0	0	0	0	0	0	0	0	0	0	0
933	0	0	0	0	0	0	0	0	9	0	0	0
934	0	8	0	0	0	0	0	0	0	0	0	0
935	0	0	0	0	0	0	0	0	0	9	0	0
936	0	0	0	0	0	0	0	0	0	0	0	0
937	0	0	0	0	0	0	0	0	0	9	0	0
938	0	0	0	0	0	0	0	0	0	9	0	0
939	0	0	0	0	0	0	0	0	9	0	0	0
940	0	0	0	0	0	0	0	0	9	0	0	0
941	0	0	0	0	0	0	0	0	9	0	0	0
942	0	0	0	0	0	0	0	0	0	0	0	0
943	0	0	0	0	0	0	0	0	9	0	0	0
944	0	0	0	0	0	0	0	0	9	0	0	0
945	0	0	0	0	0	0	0	9	0	0	0	0

OTU Number	7.11.15 Rep1	7.11.15 Rep2	7.11.15 Rep3	8.29.15 Rep1	8.29.15 Rep2	8.29.15 Rep3	2.6.16 Rep1	2.6.16 Rep2	2.6.16 Rep3	12.10.16 Rep1	12.10.16 Rep2	12.10.16 Rep3
946	0	0	0	0	0	0	0	0	9	0	0	0
947	0	0	0	0	0	0	0	0	0	0	0	0
948	0	0	0	0	0	0	0	0	9	0	0	0
949	0	0	0	0	0	0	0	9	0	0	0	0
950	0	0	0	0	0	0	0	0	9	0	0	0
951	0	0	0	0	0	0	0	0	0	9	0	0
952	0	0	0	0	0	0	0	0	0	9	0	0
953	0	0	0	0	0	0	0	0	0	0	0	0
954	0	0	0	0	0	0	0	0	0	9	0	0
955	0	0	0	0	0	0	0	0	9	0	0	0
956	0	0	0	0	0	0	0	0	0	0	0	0
957	0	0	0	0	0	0	0	0	9	0	0	0
958	0	0	0	0	0	0	0	9	0	0	0	0
959	0	0	0	0	0	0	0	0	0	0	0	0
960	0	0	0	0	0	0	0	0	0	9	0	0
961	0	0	0	0	0	0	0	0	0	9	0	0
962	0	0	0	0	0	0	0	0	9	0	0	0
963	0	0	0	0	0	0	0	9	0	0	0	0
964	0	0	0	0	0	0	0	0	0	0	0	0
965	0	0	0	0	0	0	0	9	0	0	0	0
966	0	0	0	0	0	0	0	0	0	0	0	0
967	0	0	0	0	0	0	0	9	0	0	0	0
968	0	0	0	0	0	0	0	9	0	0	0	0
969	0	0	0	0	0	0	0	9	0	0	0	0
970	2	0	0	0	0	0	0	0	0	0	0	0
971	0	0	0	0	0	0	0	0	0	9	0	0
972	0	0	0	0	0	0	0	0	0	9	0	0
973	0	0	0	0	0	0	0	0	0	9	0	0
974	0	0	0	0	0	0	0	0	0	0	0	0
975	0	0	0	0	0	0	0	0	0	0	0	0
976	0	0	0	0	0	0	0	0	9	0	0	0
977	0	0	0	0	0	0	0	0	0	0	0	0
978	0	0	0	0	0	0	0	0	9	0	0	0
979	0	0	0	0	0	0	0	0	0	0	0	9
980	0	0	0	0	0	0	0	0	0	0	0	0
981	1	0	0	0	0	1	0	0	0	0	0	0
982	0	0	0	0	0	0	0	0	0	9	0	0
983	1	2	2	0	1	0	0	0	0	0	0	0
984	0	0	0	0	0	0	0	0	9	0	0	0
985	0	0	0	0	0	0	0	9	0	0	0	0
986	0	0	0	0	0	0	0	0	0	0	0	0
987	0	0	0	0	0	0	0	0	0	0	0	9
988	0	2	1	1	2	0	0	0	0	0	0	0
989	0	0	0	0	0	0	0	0	0	9	0	0
990	0	0	0	0	0	0	0	0	0	0	9	0

OTU Number	7.11.15 Rep1	7.11.15 Rep2	7.11.15 Rep3	8.29.15 Rep1	8.29.15 Rep2	8.29.15 Rep3	2.6.16 Rep1	2.6.16 Rep2	2.6.16 Rep3	12.10.16 Rep1	12.10.16 Rep2	12.10.16 Rep3
991	0	0	0	0	0	0	0	0	9	0	0	0
992	0	0	0	0	0	0	0	0	9	0	0	0
993	1	0	0	0	0	0	0	0	0	0	0	0
994	0	0	0	0	0	0	0	9	0	0	0	0
995	0	0	0	0	0	0	0	0	9	0	0	0
996	0	0	0	0	0	0	0	0	0	0	0	0
997	0	0	0	0	0	0	0	0	9	0	0	0
998	0	0	0	0	0	0	0	0	0	9	0	0
999	0	0	0	0	0	0	0	9	0	0	0	0
1000	0	0	0	0	0	0	0	0	0	0	0	9
1001	0	0	0	0	0	0	0	0	0	0	0	0
1002	0	0	0	0	0	0	0	0	0	0	0	0
1003	0	0	0	0	0	0	0	0	0	0	0	0
1004	0	0	0	0	0	0	0	0	0	9	0	0
1005	0	0	0	0	0	0	0	0	0	0	0	0
1006	0	0	0	0	0	0	0	0	0	0	0	0
1007	0	0	0	0	0	0	0	0	0	0	0	9
1008	0	0	0	0	0	0	0	9	0	0	0	0
1009	0	0	0	0	0	0	0	0	0	0	0	0
1010	0	0	0	0	0	0	0	0	0	0	9	0
1011	0	0	0	0	0	0	0	0	0	0	9	0
1012	0	0	0	0	0	0	0	0	0	0	9	0
1013	0	0	0	0	0	7	0	0	0	0	0	0
1014	0	0	0	0	0	0	0	9	0	0	0	0
1015	0	0	0	0	0	0	0	9	0	0	0	0
1016	0	0	0	0	0	0	0	0	0	0	9	0
1017	0	0	0	0	0	0	0	9	0	0	0	0
1018	0	0	0	0	2	0	0	0	0	0	0	0
1019	0	0	0	0	0	0	0	0	0	9	0	0
1020	0	0	0	0	0	0	0	0	0	0	0	0
1021	0	0	0	0	0	0	0	0	0	0	0	0
1022	0	0	0	0	0	0	0	0	0	9	0	0
1023	0	0	0	0	0	0	0	0	0	0	0	9
1024	0	0	0	0	0	0	0	9	0	0	0	0
1025	0	0	0	0	0	0	0	0	0	0	0	0
1026	0	0	0	0	0	0	0	0	0	0	0	9
1027	0	0	0	0	0	0	0	0	0	0	0	0
1028	0	0	0	0	0	0	0	0	0	0	9	0
1029	0	0	0	0	0	0	0	9	0	0	0	0
1030	0	0	0	0	0	0	0	9	0	0	0	0
1031	0	0	0	0	0	0	0	0	0	9	0	0
1032	0	0	0	0	0	0	0	0	0	0	0	0
1033	0	0	0	0	0	0	0	0	0	0	0	0
1034	0	0	0	0	0	0	0	0	0	0	9	0
1035	0	0	0	0	0	0	0	0	0	9	0	0

OTU Number	7.11.15 Rep1	7.11.15 Rep2	7.11.15 Rep3	8.29.15 Rep1	8.29.15 Rep2	8.29.15 Rep3	2.6.16 Rep1	2.6.16 Rep2	2.6.16 Rep3	12.10.16 Rep1	12.10.16 Rep2	12.10.16 Rep3
1036	0	0	0	0	0	0	0	0	0	0	0	0
1037	0	0	0	0	0	0	0	0	9	0	0	0
1038	0	0	0	0	0	0	0	9	0	0	0	0
1039	0	0	0	0	0	0	0	0	0	0	0	9
1040	0	0	0	0	0	0	0	0	9	0	0	0
1041	0	0	0	0	0	0	0	9	0	0	0	0
1042	6	0	0	0	0	0	0	0	0	0	0	0
1043	0	0	0	0	0	0	0	0	0	0	9	0
1044	0	0	0	0	0	0	0	0	0	0	0	0
1045	0	0	0	0	0	0	0	0	0	0	9	0
1046	0	0	0	0	0	0	0	0	0	0	9	0
1047	0	0	0	0	0	0	0	0	0	0	0	9
1048	0	0	0	0	0	0	0	0	9	0	0	0
1049	0	0	0	0	0	0	0	0	9	0	0	0
1050	0	0	0	0	0	0	0	0	0	9	0	0
1051	0	0	0	0	0	0	0	0	9	0	0	0
1052	0	0	0	0	0	0	0	0	0	0	9	0
1053	0	0	0	0	0	0	0	0	0	0	9	0
1054	0	0	0	0	0	0	0	0	9	0	0	0
1055	0	0	0	0	0	0	0	0	0	9	0	0
1056	0	0	0	0	0	0	0	0	0	0	0	0
1057	0	0	0	0	0	0	0	0	0	0	0	0
1058	0	0	0	0	0	0	0	0	0	0	9	0
1059	0	0	0	0	0	0	0	0	0	9	0	0
1060	0	0	0	0	0	0	0	0	0	9	0	0
1061	0	0	0	0	0	0	0	9	0	0	0	0
1062	0	0	0	0	0	0	0	0	0	0	0	0
1063	6	0	0	0	0	0	0	0	0	0	0	0
1064	0	0	0	0	0	0	0	0	0	8	0	0
1065	0	0	0	0	0	0	0	0	0	0	0	0
1066	0	0	0	0	0	0	0	8	0	0	0	0
1067	0	0	0	0	0	0	0	0	0	0	0	8
1068	0	0	0	0	0	0	0	0	0	0	0	8
1069	0	0	0	0	0	0	0	0	0	0	0	0
1070	0	0	0	0	0	0	0	0	0	0	0	0
1071	0	0	0	0	0	0	0	8	0	0	0	0
1072	0	0	0	2	6	0	0	0	0	0	0	0
1073	0	0	0	0	4	1	0	0	0	0	0	0
1074	0	0	0	0	0	0	0	0	8	0	0	0
1075	0	0	0	0	0	0	0	0	0	8	0	0
1076	0	0	0	0	0	0	8	0	0	0	0	0
1077	0	0	0	0	0	0	0	0	8	0	0	0
1078	0	0	0	0	0	0	0	0	0	0	0	0
1079	0	0	0	0	0	0	0	0	8	0	0	0
1080	0	0	0	0	0	0	0	8	0	0	0	0

OTU Number	7.11.15 Rep1	7.11.15 Rep2	7.11.15 Rep3	8.29.15 Rep1	8.29.15 Rep2	8.29.15 Rep3	2.6.16 Rep1	2.6.16 Rep2	2.6.16 Rep3	12.10.16 Rep1	12.10.16 Rep2	12.10.16 Rep3
1081	0	0	0	0	0	0	0	0	8	0	0	0
1082	0	0	0	0	0	0	0	0	0	0	0	0
1083	0	0	0	0	0	0	0	0	0	0	8	0
1084	0	0	0	0	0	0	0	0	0	0	0	8
1085	0	0	0	0	0	0	0	8	0	0	0	0
1086	0	0	0	0	0	0	0	0	0	0	0	8
1087	0	0	0	0	0	0	0	0	0	8	0	0
1088	0	0	0	0	0	0	0	0	0	8	0	0
1089	1	0	0	2	2	0	0	0	0	0	0	0
1090	0	2	0	0	0	0	0	0	0	0	0	0
1091	0	0	0	0	0	0	0	0	0	0	0	0
1092	0	0	0	0	0	0	0	0	0	8	0	0
1093	0	0	0	0	0	0	0	0	0	8	0	0
1094	0	0	0	0	0	0	0	0	0	0	0	0
1095	0	0	0	0	0	0	0	0	0	8	0	0
1096	0	0	0	0	0	0	0	8	0	0	0	0
1097	0	0	0	0	0	0	0	0	0	0	0	0
1098	0	0	0	0	0	0	0	0	8	0	0	0
1099	0	0	0	0	0	0	0	0	0	8	0	0
1100	0	0	0	0	0	0	0	0	0	0	0	0
1101	0	0	0	0	0	0	0	0	0	8	0	0
1102	0	0	0	0	0	0	0	0	0	0	8	0
1103	0	0	0	0	0	0	0	0	0	0	8	0
1104	0	0	0	0	0	0	0	0	0	8	0	0
1105	0	0	0	0	0	0	0	0	0	0	0	0
1106	0	0	0	0	0	0	0	0	0	0	8	0
1107	0	0	0	0	0	0	0	0	0	0	0	0
1108	0	0	0	0	0	0	0	0	0	0	0	0
1109	0	0	0	0	0	0	0	0	0	0	0	0
1110	0	0	0	0	0	0	0	0	0	8	0	0
1111	0	0	0	0	0	0	0	0	0	8	0	0
1112	0	0	0	0	0	0	0	8	0	0	0	0
1113	0	0	0	0	0	0	0	8	0	0	0	0
1114	0	0	0	0	0	0	0	0	0	0	0	0
1115	0	0	0	0	0	0	0	8	0	0	0	0
1116	0	0	0	0	0	0	0	0	0	0	0	0
1117	0	0	0	0	0	0	0	8	0	0	0	0
1118	0	0	0	0	0	0	0	0	0	8	0	0
1119	0	0	0	0	0	0	0	8	0	0	0	0
1120	0	0	0	0	0	0	0	0	0	0	8	0
1121	0	0	0	0	0	0	0	8	0	0	0	0
1122	7	1	0	0	0	0	0	0	0	0	0	0
1123	0	0	0	0	0	0	0	8	0	0	0	0
1124	0	0	0	0	0	0	0	0	8	0	0	0
1125	0	0	0	0	0	0	0	0	0	0	0	0

OTU Number	7.11.15 Rep1	7.11.15 Rep2	7.11.15 Rep3	8.29.15 Rep1	8.29.15 Rep2	8.29.15 Rep3	2.6.16 Rep1	2.6.16 Rep2	2.6.16 Rep3	12.10.16 Rep1	12.10.16 Rep2	12.10.16 Rep3
1126	0	0	0	0	0	0	0	8	0	0	0	0
1127	0	0	0	0	0	0	0	8	0	0	0	0
1128	0	0	0	0	0	0	0	0	0	8	0	0
1129	0	0	0	0	0	0	0	0	8	0	0	0
1130	2	2	0	0	0	0	0	0	0	0	0	0
1131	0	0	0	0	0	0	0	0	8	0	0	0
1132	0	0	0	0	0	0	0	0	0	0	0	0
1133	0	0	0	0	0	0	0	0	0	0	0	0
1134	0	1	0	0	0	0	0	0	0	0	0	0
1135	0	0	0	0	0	0	0	0	8	0	0	0
1136	0	0	0	0	0	0	0	0	0	0	0	0
1137	0	0	0	0	0	0	0	8	0	0	0	0
1138	0	0	0	0	0	0	0	8	0	0	0	0
1139	0	0	0	0	0	0	0	0	0	0	0	0
1140	0	0	0	0	0	0	0	8	0	0	0	0
1141	0	0	0	0	0	0	0	0	0	8	0	0
1142	0	0	0	0	0	0	0	0	0	0	0	0
1143	0	0	0	0	0	0	0	0	0	0	0	0
1144	0	0	0	0	0	0	0	0	8	0	0	0
1145	0	0	0	0	0	0	0	8	0	0	0	0
1146	0	0	0	0	0	0	0	0	0	8	0	0
1147	0	0	0	0	0	0	0	0	0	8	0	0
1148	0	0	0	0	0	0	0	0	0	8	0	0
1149	0	0	0	0	0	0	0	0	8	0	0	0
1150	0	0	0	0	0	0	0	0	0	0	0	0
1151	0	0	0	0	0	0	0	0	0	8	0	0
1152	0	0	0	0	0	0	0	8	0	0	0	0
1153	0	0	0	0	0	0	0	0	0	8	0	0
1154	0	0	0	0	0	0	0	0	0	0	0	8
1155	0	0	0	0	0	0	0	0	0	0	0	0
1156	0	0	0	0	0	0	0	0	0	0	8	0
1157	0	0	0	0	0	0	0	0	0	0	0	0
1158	0	0	0	0	0	0	0	8	0	0	0	0
1159	0	0	0	0	0	0	0	0	0	0	0	8
1160	0	0	0	0	0	0	0	0	0	0	0	0
1161	0	0	0	0	0	0	0	0	0	0	0	0
1162	4	0	0	0	0	0	0	0	0	0	0	0
1163	0	0	0	0	0	0	0	0	0	8	0	0
1164	0	1	0	0	0	0	0	0	0	0	0	0
1165	0	0	0	0	0	0	0	0	0	0	0	0
1166	0	0	0	0	0	0	0	0	0	0	8	0
1167	0	0	0	0	0	0	0	0	0	0	0	0
1168	0	0	0	0	0	0	6	0	0	0	0	0
1169	0	0	0	0	0	0	0	0	0	0	0	0
1170	0	0	0	0	0	0	0	0	0	0	0	0

OTU Number	7.11.15 Rep1	7.11.15 Rep2	7.11.15 Rep3	8.29.15 Rep1	8.29.15 Rep2	8.29.15 Rep3	2.6.16 Rep1	2.6.16 Rep2	2.6.16 Rep3	12.10.16 Rep1	12.10.16 Rep2	12.10.16 Rep3
1171	0	0	0	0	0	0	0	0	0	0	0	8
1172	0	0	0	0	0	0	0	0	8	0	0	0
1173	0	0	0	0	0	0	0	0	0	0	8	0
1174	0	0	0	0	0	0	0	0	0	8	0	0
1175	0	0	0	0	0	0	0	0	0	0	0	0
1176	4	0	0	0	2	0	0	0	0	0	0	0
1177	1	5	0	0	0	0	0	0	0	0	0	0
1178	0	0	0	0	0	0	0	0	0	0	0	0
1179	0	0	0	0	2	6	0	0	0	0	0	0
1180	0	1	0	0	0	0	0	0	0	0	0	0
1181	0	0	0	0	0	0	0	0	0	0	0	0
1182	0	0	0	0	1	0	1	0	0	0	0	0
1183	0	0	2	4	1	1	0	0	0	0	0	0
1184	0	0	0	0	0	0	0	0	0	0	0	0
1185	2	0	1	0	0	0	0	0	0	0	0	0
1186	0	0	0	0	0	0	0	0	0	0	8	0
1187	0	0	0	0	0	0	0	0	8	0	0	0
1188	0	0	0	0	0	0	0	0	0	0	0	8
1189	0	0	0	0	0	0	0	0	0	8	0	0
1190	0	0	0	0	0	0	0	0	0	0	0	8
1191	0	0	0	0	0	0	0	0	0	0	0	8
1192	0	0	0	0	0	0	0	0	0	8	0	0
1193	0	0	0	0	0	0	0	0	0	0	8	0
1194	0	0	0	0	0	0	0	8	0	0	0	0
1195	0	0	0	0	0	0	0	0	0	8	0	0
1196	0	0	0	0	0	0	0	0	0	0	8	0
1197	0	0	0	0	0	0	0	8	0	0	0	0
1198	0	0	0	0	0	0	0	0	0	0	0	8
1199	0	0	0	0	0	0	0	0	0	0	0	8
1200	0	0	0	0	0	0	0	0	0	0	0	0
1201	0	0	0	0	0	0	0	0	0	0	0	8
1202	0	0	0	0	0	0	0	8	0	0	0	0
1203	0	0	0	0	0	0	0	0	0	0	0	0
1204	0	0	0	0	0	0	0	0	8	0	0	0
1205	0	0	0	0	0	0	0	0	0	0	0	8
1206	0	0	0	0	0	0	0	0	0	0	0	0
1207	0	0	0	0	0	0	0	0	0	0	0	0
1208	0	0	0	0	0	0	0	0	0	0	0	0
1209	0	0	0	0	0	0	0	0	0	0	0	8
1210	0	0	0	0	0	0	0	0	0	0	0	8
1211	0	0	0	0	0	0	0	0	8	0	0	0
1212	0	0	0	0	0	0	0	0	0	0	8	0
1213	0	0	0	0	0	0	0	0	0	0	0	8
1214	0	0	0	0	0	0	0	0	0	0	0	0
1215	0	0	0	0	0	0	0	8	0	0	0	0

OTU Number	7.11.15 Rep1	7.11.15 Rep2	7.11.15 Rep3	8.29.15 Rep1	8.29.15 Rep2	8.29.15 Rep3	2.6.16 Rep1	2.6.16 Rep2	2.6.16 Rep3	12.10.16 Rep1	12.10.16 Rep2	12.10.16 Rep3
1216	0	0	0	0	0	0	0	0	0	0	0	0
1217	0	0	0	0	0	0	0	0	0	0	8	0
1218	0	0	0	0	0	0	0	0	7	0	0	0
1219	0	0	0	0	0	0	0	0	0	7	0	0
1220	0	0	0	0	0	0	0	0	0	0	7	0
1221	0	0	0	0	0	0	0	0	7	0	0	0
1222	0	0	0	0	0	0	0	0	0	7	0	0
1223	0	0	0	0	0	0	0	0	0	0	0	7
1224	0	0	0	0	0	0	0	7	0	0	0	0
1225	0	0	0	0	0	0	0	0	0	7	0	0
1226	0	0	0	0	0	0	0	0	0	0	0	7
1227	0	0	0	0	0	0	0	0	7	0	0	0
1228	0	0	0	0	0	0	0	0	0	0	0	0
1229	0	0	0	0	0	0	2	0	0	0	0	0
1230	0	0	0	0	0	0	0	0	0	0	0	0
1231	0	1	0	0	0	0	0	0	0	0	0	0
1232	0	0	0	0	0	0	0	0	0	0	0	0
1233	0	0	0	0	0	0	0	7	0	0	0	0
1234	0	0	0	0	0	0	0	0	7	0	0	0
1235	0	0	0	0	0	0	0	0	0	0	0	0
1236	0	0	0	0	0	0	0	0	0	0	7	0
1237	0	0	0	0	0	0	0	0	0	0	0	0
1238	0	0	0	0	0	0	0	7	0	0	0	0
1239	0	0	0	0	0	0	0	0	0	0	7	0
1240	0	0	0	0	0	0	0	0	7	0	0	0
1241	0	0	0	0	0	0	0	0	0	7	0	0
1242	0	0	0	0	0	0	0	0	0	0	0	0
1243	0	0	0	0	0	0	0	7	0	0	0	0
1244	0	0	0	0	0	0	0	0	0	0	7	0
1245	0	0	0	0	0	0	0	0	0	7	0	0
1246	0	0	0	0	0	0	0	0	0	0	0	0
1247	0	0	0	0	0	0	0	0	0	0	7	0
1248	0	0	0	0	0	0	0	0	0	0	7	0
1249	0	0	0	0	0	0	0	0	0	7	0	0
1250	0	0	0	0	0	0	0	0	0	7	0	0
1251	0	0	0	0	0	0	0	0	0	0	0	0
1252	0	0	0	0	0	0	0	0	7	0	0	0
1253	0	0	0	0	0	0	0	0	0	7	0	0
1254	0	0	0	0	0	0	0	0	0	0	0	0
1255	0	0	0	0	0	0	0	0	0	0	7	0
1256	0	0	0	0	0	0	0	0	0	0	0	0
1257	0	0	0	0	0	0	0	0	0	7	0	0
1258	0	3	0	0	0	0	0	0	0	0	0	0
1259	0	0	0	0	0	0	0	7	0	0	0	0
1260	0	0	0	0	0	0	0	0	7	0	0	0

OTU Number	7.11.15 Rep1	7.11.15 Rep2	7.11.15 Rep3	8.29.15 Rep1	8.29.15 Rep2	8.29.15 Rep3	2.6.16 Rep1	2.6.16 Rep2	2.6.16 Rep3	12.10.16 Rep1	12.10.16 Rep2	12.10.16 Rep3
1261	0	0	0	0	0	0	0	0	7	0	0	0
1262	1	5	1	0	0	0	0	0	0	0	0	0
1263	0	0	0	0	0	0	0	0	7	0	0	0
1264	0	0	0	0	0	0	0	0	0	7	0	0
1265	0	0	0	0	0	0	0	7	0	0	0	0
1266	0	0	0	0	0	0	0	0	0	0	0	7
1267	0	0	0	0	0	0	0	0	0	7	0	0
1268	0	0	0	0	0	0	0	0	0	0	0	7
1269	0	0	0	0	0	0	7	0	0	0	0	0
1270	0	0	0	0	0	0	0	0	0	0	7	0
1271	0	0	0	0	0	0	0	0	0	7	0	0
1272	0	0	0	0	0	0	0	0	0	0	7	0
1273	0	0	0	0	0	0	0	0	0	0	0	0
1274	0	0	0	0	0	0	0	0	0	0	0	7
1275	0	0	0	0	0	0	0	0	0	0	0	0
1276	0	0	0	0	0	0	0	7	0	0	0	0
1277	0	0	0	0	0	0	0	0	0	0	0	7
1278	0	0	0	0	0	0	0	0	0	0	0	7
1279	0	0	0	0	0	0	0	7	0	0	0	0
1280	0	0	0	0	0	0	0	0	7	0	0	0
1281	0	0	0	0	0	0	0	0	7	0	0	0
1282	0	0	0	0	0	0	0	7	0	0	0	0
1283	0	0	0	0	0	0	0	0	0	0	0	7
1284	0	0	0	0	0	0	0	7	0	0	0	0
1285	0	0	0	0	0	0	0	0	0	0	0	0
1286	0	0	0	0	4	0	0	0	0	0	0	0
1287	0	0	0	0	0	0	0	0	0	0	7	0
1288	0	0	0	0	0	0	0	7	0	0	0	0
1289	0	0	0	0	0	0	0	0	0	7	0	0
1290	0	0	0	0	0	0	0	0	0	0	0	0
1291	0	0	0	0	0	0	0	0	0	7	0	0
1292	0	0	0	0	0	0	0	0	0	0	0	7
1293	0	0	0	0	0	0	0	0	0	0	0	0
1294	0	0	0	0	0	0	0	0	0	0	7	0
1295	0	0	0	0	0	0	0	0	0	7	0	0
1296	0	0	0	0	0	0	0	0	7	0	0	0
1297	0	0	0	0	0	0	0	0	0	0	0	0
1298	0	0	0	0	0	0	0	0	0	0	0	0
1299	1	0	0	0	0	0	0	0	0	0	0	0
1300	0	0	0	0	0	0	0	0	0	0	0	7
1301	0	0	0	0	0	0	0	0	0	0	0	0
1302	0	0	0	0	0	0	0	0	0	0	0	0
1303	0	0	0	0	0	0	0	0	0	0	0	0
1304	0	0	0	0	0	0	0	0	0	0	7	0
1305	0	0	0	0	0	0	0	0	0	7	0	0

OTU Number	7.11.15 Rep1	7.11.15 Rep2	7.11.15 Rep3	8.29.15 Rep1	8.29.15 Rep2	8.29.15 Rep3	2.6.16 Rep1	2.6.16 Rep2	2.6.16 Rep3	12.10.16 Rep1	12.10.16 Rep2	12.10.16 Rep3
1306	0	0	0	0	0	0	0	0	0	0	0	0
1307	0	0	0	0	0	0	0	7	0	0	0	0
1308	0	0	0	0	0	0	0	0	7	0	0	0
1309	0	0	0	0	0	0	0	0	0	0	0	0
1310	0	0	0	0	0	0	0	0	0	0	0	0
1311	0	0	0	0	0	0	0	7	0	0	0	0
1312	0	0	0	0	0	0	0	0	0	7	0	0
1313	0	0	0	0	0	0	0	0	0	0	0	0
1314	0	0	0	0	0	0	0	0	0	0	7	0
1315	0	0	0	0	0	0	0	0	7	0	0	0
1316	0	0	0	0	0	0	0	7	0	0	0	0
1317	0	0	0	0	0	0	0	0	7	0	0	0
1318	0	0	0	0	0	0	0	7	0	0	0	0
1319	0	0	0	0	0	0	0	0	7	0	0	0
1320	0	0	0	0	0	0	0	0	7	0	0	0
1321	0	0	0	0	0	0	0	0	0	0	0	7
1322	0	0	0	0	0	0	0	0	0	0	7	0
1323	0	0	0	0	0	0	0	0	0	0	0	0
1324	0	1	0	0	0	0	0	0	0	0	0	0
1325	0	0	0	0	0	0	0	0	0	0	0	7
1326	0	0	0	0	0	0	0	0	7	0	0	0
1327	0	0	0	0	0	0	0	0	7	0	0	0
1328	0	0	0	0	0	0	0	0	0	0	0	7
1329	0	0	0	0	0	0	0	0	0	0	7	0
1330	0	0	0	0	0	0	0	0	0	0	7	0
1331	0	0	0	0	0	0	0	0	0	0	0	0
1332	0	0	0	0	0	0	0	0	0	7	0	0
1333	0	0	0	0	1	0	0	0	0	0	0	0
1334	0	0	0	0	0	0	0	0	0	7	0	0
1335	0	0	0	0	0	0	0	0	7	0	0	0
1336	0	0	0	0	0	0	0	0	0	0	0	0
1337	0	0	0	0	0	0	0	0	0	7	0	0
1338	0	0	0	0	0	0	0	0	7	0	0	0
1339	0	0	0	0	0	0	0	0	0	7	0	0
1340	0	0	0	0	0	0	0	0	0	0	0	7
1341	1	0	0	1	4	0	0	0	0	0	0	0
1342	0	0	0	0	0	0	0	0	0	0	7	0
1343	0	0	0	0	0	0	0	0	0	0	0	0
1344	0	0	0	0	0	0	0	0	0	0	0	0
1345	0	0	0	0	0	0	0	7	0	0	0	0
1346	0	0	0	0	0	0	0	7	0	0	0	0
1347	0	0	0	0	0	0	0	7	0	0	0	0
1348	0	0	0	0	0	0	0	0	0	0	7	0
1349	0	0	0	0	1	0	0	0	0	0	0	0
1350	0	0	0	0	0	0	0	0	0	0	0	0

OTU Number	7.11.15 Rep1	7.11.15 Rep2	7.11.15 Rep3	8.29.15 Rep1	8.29.15 Rep2	8.29.15 Rep3	2.6.16 Rep1	2.6.16 Rep2	2.6.16 Rep3	12.10.16 Rep1	12.10.16 Rep2	12.10.16 Rep3
1351	0	0	0	0	0	0	0	7	0	0	0	0
1352	0	0	0	0	0	0	0	7	0	0	0	0
1353	0	0	0	0	0	0	0	7	0	0	0	0
1354	0	0	0	0	0	0	0	7	0	0	0	0
1355	2	0	1	0	0	0	0	0	0	0	0	0
1356	0	0	0	0	0	0	0	0	7	0	0	0
1357	0	0	0	0	0	0	0	0	0	0	0	0
1358	0	0	0	0	0	0	0	0	0	7	0	0
1359	0	0	0	0	0	0	0	0	7	0	0	0
1360	0	0	0	0	0	0	0	0	7	0	0	0
1361	0	0	0	0	0	0	0	0	0	7	0	0
1362	0	0	0	0	0	0	0	7	0	0	0	0
1363	0	0	0	0	0	0	0	0	7	0	0	0
1364	0	0	0	0	0	0	0	0	0	0	0	7
1365	0	0	0	0	0	0	0	0	0	7	0	0
1366	0	0	0	0	0	0	0	0	7	0	0	0
1367	0	0	0	0	0	0	0	0	7	0	0	0
1368	1	0	0	1	1	1	0	0	0	0	0	0
1369	0	0	0	0	0	0	0	0	0	0	0	0
1370	0	0	0	0	0	0	0	7	0	0	0	0
1371	0	0	0	0	0	0	0	0	0	0	6	1
1372	0	0	0	0	0	0	0	7	0	0	0	0
1373	0	0	0	0	0	0	0	0	0	0	0	0
1374	0	0	0	0	3	2	0	0	0	0	0	0
1375	0	0	0	0	0	0	0	7	0	0	0	0
1376	0	0	0	0	0	0	0	0	0	7	0	0
1377	0	0	0	0	0	0	0	0	0	0	7	0
1378	0	0	0	0	0	0	0	7	0	0	0	0
1379	0	0	0	1	6	0	0	0	0	0	0	0
1380	0	0	0	0	0	0	0	0	0	7	0	0
1381	0	0	0	0	0	0	0	0	0	0	0	0
1382	0	0	0	0	0	0	0	0	0	7	0	0
1383	1	0	0	0	0	0	0	0	0	0	0	0
1384	0	0	0	0	0	0	0	7	0	0	0	0
1385	0	0	0	0	0	0	0	7	0	0	0	0
1386	0	0	0	0	0	0	0	0	0	0	7	0
1387	0	0	0	2	2	0	0	0	0	0	0	0
1388	1	0	2	0	1	0	0	0	0	0	0	0
1389	0	0	0	0	0	0	0	7	0	0	0	0
1390	0	0	0	0	0	0	0	7	0	0	0	0
1391	0	0	0	0	0	0	0	7	0	0	0	0
1392	0	0	0	0	0	0	0	0	0	0	0	0
1393	0	0	0	0	0	0	0	7	0	0	0	0
1394	7	0	0	0	0	0	0	0	0	0	0	0
1395	2	2	0	0	0	0	0	0	0	0	0	0

OTU Number	7.11.15 Rep1	7.11.15 Rep2	7.11.15 Rep3	8.29.15 Rep1	8.29.15 Rep2	8.29.15 Rep3	2.6.16 Rep1	2.6.16 Rep2	2.6.16 Rep3	12.10.16 Rep1	12.10.16 Rep2	12.10.16 Rep3
1396	0	0	0	0	0	0	0	0	0	0	0	0
1397	0	0	0	0	0	0	0	0	0	0	0	7
1398	2	0	0	0	0	0	0	0	0	0	0	0
1399	0	0	0	0	0	0	0	0	0	7	0	0
1400	0	0	0	0	0	0	0	0	0	0	7	0
1401	0	0	0	0	0	0	0	7	0	0	0	0
1402	0	0	0	0	0	0	0	0	0	0	0	0
1403	0	0	0	0	0	0	0	7	0	0	0	0
1404	0	0	0	0	0	0	0	7	0	0	0	0
1405	0	0	0	0	0	0	0	7	0	0	0	0
1406	0	0	0	0	0	0	0	7	0	0	0	0
1407	0	0	0	0	0	0	0	0	0	0	0	7
1408	0	0	0	0	0	0	0	0	0	0	0	7
1409	0	0	0	0	0	0	0	0	0	7	0	0
1410	0	0	0	0	0	0	0	0	0	0	0	0
1411	0	0	0	0	0	0	0	0	0	0	0	7
1412	0	0	0	0	0	0	0	0	0	0	7	0
1413	0	0	0	0	0	0	0	7	0	0	0	0
1414	0	0	0	0	0	0	0	0	0	0	7	0
1415	0	0	0	0	1	0	0	0	0	0	0	0
1416	0	0	0	0	0	0	0	0	0	7	0	0
1417	0	0	0	0	0	0	0	0	0	0	7	0
1418	0	0	0	0	0	0	0	0	0	0	0	0
1419	0	0	0	0	0	0	0	7	0	0	0	0
1420	0	0	0	0	0	0	0	0	0	7	0	0
1421	0	0	0	0	0	0	0	7	0	0	0	0
1422	0	0	0	0	0	0	0	0	0	7	0	0
1423	0	0	0	0	0	0	0	0	0	0	0	7
1424	0	0	0	0	0	0	0	0	0	0	0	0
1425	0	0	0	0	0	0	0	0	0	7	0	0
1426	0	0	0	0	0	0	0	0	0	7	0	0
1427	0	0	0	0	0	0	0	0	0	7	0	0
1428	0	0	0	0	0	0	0	0	0	7	0	0
1429	0	0	0	0	0	0	0	0	7	0	0	0
1430	0	0	0	0	0	0	0	0	0	7	0	0
1431	0	0	0	0	0	0	0	0	0	0	0	0
1432	0	0	0	0	0	0	0	0	0	0	0	7
1433	0	0	0	0	0	0	0	7	0	0	0	0
1434	0	0	0	0	0	0	0	0	0	0	0	0
1435	0	0	0	0	0	0	0	6	0	0	0	0
1436	0	0	3	2	0	1	0	0	0	0	0	0
1437	0	0	0	0	0	0	0	0	0	0	0	0
1438	0	0	0	0	0	0	0	0	0	0	0	6
1439	0	0	0	0	0	0	0	0	6	0	0	0
1440	0	0	0	0	0	0	0	0	0	0	0	0

OTU Number	7.11.15 Rep1	7.11.15 Rep2	7.11.15 Rep3	8.29.15 Rep1	8.29.15 Rep2	8.29.15 Rep3	2.6.16 Rep1	2.6.16 Rep2	2.6.16 Rep3	12.10.16 Rep1	12.10.16 Rep2	12.10.16 Rep3
1441	0	0	0	0	0	0	0	0	0	0	0	6
1442	1	0	0	0	0	0	0	0	0	0	0	0
1443	2	0	4	0	0	0	0	0	0	0	0	0
1444	0	0	0	2	0	0	0	0	0	0	0	0
1445	0	0	1	1	3	1	0	0	0	0	0	0
1446	0	0	0	0	0	0	0	6	0	0	0	0
1447	0	0	0	0	0	0	0	0	6	0	0	0
1448	1	0	3	1	1	0	0	0	0	0	0	0
1449	0	0	0	0	0	0	0	0	0	0	0	6
1450	0	0	0	0	0	0	0	6	0	0	0	0
1451	0	0	0	0	0	0	0	0	0	0	0	0
1452	0	0	0	0	0	0	0	0	6	0	0	0
1453	0	0	0	0	0	0	0	0	6	0	0	0
1454	0	0	0	0	0	0	0	0	0	0	0	0
1455	0	0	0	0	0	0	0	0	6	0	0	0
1456	0	0	0	0	0	0	0	0	0	0	0	0
1457	0	0	0	0	0	0	0	0	6	0	0	0
1458	0	0	0	0	0	0	0	0	0	0	0	6
1459	2	0	0	0	0	0	0	0	0	0	0	0
1460	2	0	0	0	0	0	0	0	0	0	0	0
1461	0	0	0	0	0	0	0	0	6	0	0	0
1462	0	0	0	0	0	0	0	0	0	0	0	0
1463	0	0	0	0	0	0	0	0	6	0	0	0
1464	0	0	0	0	0	0	0	0	6	0	0	0
1465	0	0	0	0	0	0	0	0	0	0	0	0
1466	0	0	0	0	0	0	0	0	6	0	0	0
1467	0	0	0	0	0	0	0	0	0	0	0	0
1468	0	0	0	0	0	0	0	6	0	0	0	0
1469	0	0	0	0	0	0	0	6	0	0	0	0
1470	0	0	0	0	0	0	0	0	6	0	0	0
1471	0	0	0	0	0	0	0	0	0	0	0	6
1472	1	0	0	0	0	0	0	0	0	0	0	0
1473	0	0	0	0	0	0	0	0	0	0	6	0
1474	0	0	0	0	0	0	0	0	0	0	0	6
1475	0	0	0	0	0	0	0	0	0	0	0	0
1476	0	0	0	0	0	0	0	0	0	0	0	6
1477	0	0	0	0	0	0	0	0	0	0	0	0
1478	0	0	0	0	0	0	0	6	0	0	0	0
1479	0	0	0	0	0	0	0	0	0	0	0	0
1480	0	0	0	0	0	0	0	0	0	0	0	0
1481	0	0	0	0	0	0	0	0	6	0	0	0
1482	0	0	0	0	0	0	0	0	0	0	0	0
1483	0	0	0	0	0	0	0	0	0	0	0	0
1484	0	0	0	0	0	0	0	0	0	0	0	0
1485	0	0	0	0	0	0	0	6	0	0	0	0

OTU Number	7.11.15 Rep1	7.11.15 Rep2	7.11.15 Rep3	8.29.15 Rep1	8.29.15 Rep2	8.29.15 Rep3	2.6.16 Rep1	2.6.16 Rep2	2.6.16 Rep3	12.10.16 Rep1	12.10.16 Rep2	12.10.16 Rep3
1486	0	0	0	0	0	0	0	0	0	0	6	0
1487	0	0	0	0	0	0	0	0	0	0	0	6
1488	0	1	0	0	0	0	0	0	0	0	0	0
1489	1	0	0	0	0	0	0	0	0	0	0	0
1490	0	0	0	0	0	0	0	0	0	0	6	0
1491	3	0	0	0	0	0	0	0	0	0	0	0
1492	0	0	0	0	0	0	0	0	0	0	0	0
1493	0	0	0	0	0	0	0	6	0	0	0	0
1494	0	0	0	0	0	0	0	0	0	0	6	0
1495	0	0	0	0	0	0	0	0	6	0	0	0
1496	1	0	3	0	0	0	0	0	0	0	0	0
1497	0	0	0	0	0	0	0	0	0	0	6	0
1498	0	0	0	0	0	0	0	0	0	0	0	0
1499	0	0	0	0	0	0	0	0	0	0	0	0
1500	0	0	0	0	0	0	0	0	0	0	0	0
1501	0	0	0	0	0	0	0	0	0	0	0	0
1502	0	0	0	0	0	0	0	0	0	0	0	6
1503	0	0	0	0	1	2	0	0	0	0	0	0
1504	0	0	0	0	0	0	0	0	0	0	0	6
1505	0	0	0	0	0	0	0	6	0	0	0	0
1506	0	0	0	0	0	0	0	0	0	0	0	6
1507	0	0	0	0	0	0	0	0	0	0	6	0
1508	0	0	0	0	0	0	0	0	0	0	0	0
1509	0	0	0	0	0	0	0	6	0	0	0	0
1510	0	0	0	0	0	0	0	0	6	0	0	0
1511	0	0	0	0	0	0	0	6	0	0	0	0
1512	0	0	0	0	0	0	0	0	0	0	0	6
1513	3	1	1	0	0	0	0	0	0	0	0	0
1514	0	0	0	0	0	0	0	0	0	0	0	6
1515	0	0	0	0	0	0	0	6	0	0	0	0
1516	0	0	0	0	0	0	0	0	0	0	0	0
1517	0	0	0	0	0	0	0	0	0	0	0	0
1518	0	0	0	0	0	0	0	6	0	0	0	0
1519	0	0	0	0	0	0	0	0	0	0	0	0
1520	0	0	0	0	0	0	0	0	6	0	0	0
1521	0	0	0	0	0	0	0	0	0	0	0	0
1522	0	1	0	0	0	0	0	0	0	0	0	0
1523	0	0	0	0	0	0	0	6	0	0	0	0
1524	0	0	0	0	0	0	0	0	6	0	0	0
1525	0	0	0	0	0	0	0	0	6	0	0	0
1526	0	0	0	0	0	0	0	0	0	0	0	6
1527	0	0	0	0	0	0	0	0	0	0	0	6
1528	0	0	0	0	0	0	0	0	0	0	0	0
1529	0	3	0	0	0	0	0	0	0	0	0	0
1530	0	0	0	0	0	0	0	0	0	0	0	6

OTU Number	7.11.15 Rep1	7.11.15 Rep2	7.11.15 Rep3	8.29.15 Rep1	8.29.15 Rep2	8.29.15 Rep3	2.6.16 Rep1	2.6.16 Rep2	2.6.16 Rep3	12.10.16 Rep1	12.10.16 Rep2	12.10.16 Rep3
1531	3	0	1	0	0	0	0	0	0	0	0	0
1532	0	0	0	0	0	0	0	0	6	0	0	0
1533	0	0	0	0	0	0	0	6	0	0	0	0
1534	0	0	0	0	0	0	0	6	0	0	0	0
1535	0	0	0	0	0	0	0	0	0	0	0	0
1536	0	0	0	0	0	0	0	0	6	0	0	0
1537	0	0	0	0	0	0	0	0	0	0	0	6
1538	3	0	0	0	0	0	0	0	0	0	0	0
1539	0	0	0	0	0	0	0	6	0	0	0	0
1540	0	0	0	1	0	2	0	0	0	0	0	0
1541	0	0	0	0	0	0	0	0	0	0	0	6
1542	0	0	0	0	0	0	0	6	0	0	0	0
1543	0	2	0	1	0	0	0	0	0	0	0	0
1544	0	0	0	0	0	0	0	0	6	0	0	0
1545	0	0	0	0	0	0	0	0	0	0	0	0
1546	0	0	0	0	0	0	0	6	0	0	0	0
1547	0	0	0	0	0	0	0	0	6	0	0	0
1548	0	0	0	0	0	0	0	0	6	0	0	0
1549	0	0	0	0	0	0	0	6	0	0	0	0
1550	0	3	0	0	0	0	0	0	0	0	0	0
1551	0	0	0	0	0	0	0	6	0	0	0	0
1552	0	0	0	0	0	0	0	0	0	0	0	6
1553	0	5	0	0	0	0	0	0	0	0	0	0
1554	1	0	1	1	0	0	0	0	0	0	0	0
1555	0	0	0	0	0	0	0	6	0	0	0	0
1556	0	0	0	0	0	0	0	0	0	0	0	6
1557	0	0	0	0	0	0	0	0	6	0	0	0
1558	0	0	0	0	0	0	0	6	0	0	0	0
1559	0	0	0	0	0	0	0	0	6	0	0	0
1560	0	2	0	0	0	0	0	0	0	0	0	0
1561	0	0	0	0	0	0	0	6	0	0	0	0
1562	0	0	0	0	0	0	0	6	0	0	0	0
1563	0	0	0	0	0	0	0	6	0	0	0	0
1564	0	0	0	0	0	0	0	0	0	0	0	0
1565	0	0	0	0	0	0	0	0	0	0	0	0
1566	0	0	0	0	0	0	0	6	0	0	0	0
1567	0	6	0	0	0	0	0	0	0	0	0	0
1568	0	0	0	0	0	0	0	0	0	0	0	6
1569	0	0	0	0	0	0	0	0	0	0	0	0
1570	0	0	0	0	0	0	0	6	0	0	0	0
1571	0	0	0	0	0	0	0	0	0	0	0	6
1572	0	0	0	0	0	0	0	0	6	0	0	0
1573	0	0	0	0	0	0	0	0	0	0	0	6
1574	0	0	0	0	0	0	0	0	0	0	0	6
1575	0	0	0	0	0	0	0	0	0	0	0	6

OTU Number	7.11.15 Rep1	7.11.15 Rep2	7.11.15 Rep3	8.29.15 Rep1	8.29.15 Rep2	8.29.15 Rep3	2.6.16 Rep1	2.6.16 Rep2	2.6.16 Rep3	12.10.16 Rep1	12.10.16 Rep2	12.10.16 Rep3
1576	0	0	0	0	0	0	0	0	0	0	0	6
1577	0	0	0	0	0	0	0	6	0	0	0	0
1578	0	0	0	0	0	0	0	0	0	0	0	6
1579	0	0	0	0	0	0	0	6	0	0	0	0
1580	0	0	0	0	0	0	0	0	6	0	0	0
1581	0	0	0	0	0	0	0	0	6	0	0	0
1582	0	0	0	0	0	0	0	0	6	0	0	0
1583	0	0	0	0	3	0	0	0	0	0	0	0
1584	0	0	0	0	0	0	0	0	0	0	0	6
1585	0	0	0	0	0	0	0	0	0	0	0	6
1586	0	0	0	0	0	0	0	0	0	0	0	6
1587	0	0	0	0	0	0	3	0	0	0	0	0
1588	0	0	0	0	0	0	0	6	0	0	0	0
1589	0	1	0	0	0	0	0	0	0	0	0	0
1590	0	0	0	0	0	0	0	0	0	0	0	0
1591	0	0	0	2	0	0	0	0	0	0	0	0
1592	0	0	0	0	0	0	0	0	6	0	0	0
1593	0	0	0	0	0	0	0	6	0	0	0	0
1594	0	0	0	0	0	0	0	6	0	0	0	0
1595	0	0	0	0	0	0	0	6	0	0	0	0
1596	0	0	0	0	0	0	0	0	6	0	0	0
1597	0	0	0	0	0	0	0	0	0	0	0	6
1598	0	0	0	0	0	0	0	6	0	0	0	0
1599	0	0	0	0	0	0	0	0	6	0	0	0
1600	0	0	0	0	0	0	0	0	0	0	0	6
1601	0	6	0	0	0	0	0	0	0	0	0	0
1602	0	0	0	0	0	0	0	0	0	0	0	6
1603	0	0	0	0	0	0	0	6	0	0	0	0
1604	0	0	0	0	0	0	0	0	6	0	0	0
1605	0	0	0	0	0	0	0	0	0	0	0	6
1606	0	0	0	0	0	0	0	0	6	0	0	0
1607	0	0	0	0	0	0	0	0	6	0	0	0
1608	0	0	0	0	0	0	0	6	0	0	0	0
1609	0	0	0	0	0	0	0	0	6	0	0	0
1610	0	0	0	0	0	0	0	0	6	0	0	0
1611	0	0	0	0	0	0	0	0	0	0	0	0
1612	0	0	0	0	0	0	0	0	0	6	0	0
1613	0	0	0	0	0	0	0	0	0	6	0	0
1614	0	0	0	0	0	0	0	0	0	6	0	0
1615	0	0	0	0	0	0	0	0	0	6	0	0
1616	0	0	0	0	0	0	0	0	0	0	0	0
1617	0	0	0	0	0	0	0	0	0	6	0	0
1618	0	0	0	0	0	0	0	0	0	0	6	0
1619	0	0	0	0	0	0	0	0	0	0	6	0
1620	0	0	0	0	0	0	0	0	0	0	0	0

OTU Number	7.11.15 Rep1	7.11.15 Rep2	7.11.15 Rep3	8.29.15 Rep1	8.29.15 Rep2	8.29.15 Rep3	2.6.16 Rep1	2.6.16 Rep2	2.6.16 Rep3	12.10.16 Rep1	12.10.16 Rep2	12.10.16 Rep3
1621	0	0	0	0	0	0	0	0	0	6	0	0
1622	0	0	0	0	0	0	0	0	0	0	6	0
1623	0	0	0	0	0	0	0	0	0	0	0	0
1624	0	0	0	0	0	0	0	0	0	0	0	0
1625	0	0	0	0	0	0	0	0	0	0	0	0
1626	0	0	0	0	0	0	0	0	0	0	0	0
1627	0	0	0	0	0	0	0	0	0	0	6	0
1628	0	0	0	0	0	0	0	0	0	0	0	0
1629	0	0	0	0	0	0	0	0	0	0	0	0
1630	0	0	0	0	0	0	0	0	0	0	6	0
1631	0	0	0	0	0	0	0	0	0	6	0	0
1632	0	0	0	0	0	0	0	0	0	6	0	0
1633	0	0	0	0	0	0	0	0	0	6	0	0
1634	0	0	0	0	0	0	0	0	0	6	0	0
1635	0	0	0	0	0	0	0	0	0	6	0	0
1636	0	0	0	0	0	0	0	0	0	6	0	0
1637	0	0	0	0	0	0	0	0	0	6	0	0
1638	0	0	0	0	0	0	0	0	0	6	0	0
1639	0	0	0	0	0	0	0	0	0	6	0	0
1640	0	0	0	0	0	0	0	0	0	6	0	0
1641	0	0	0	0	0	0	0	0	0	0	0	0
1642	0	0	0	0	0	0	0	0	0	6	0	0
1643	0	0	0	0	0	0	0	0	0	0	0	0
1644	0	0	0	0	0	0	0	0	0	6	0	0
1645	0	0	0	0	0	0	0	0	0	6	0	0
1646	0	0	0	0	0	0	0	0	0	6	0	0
1647	0	0	0	0	0	0	0	0	0	0	6	0
1648	0	0	0	0	0	0	0	0	0	6	0	0
1649	0	0	0	0	0	0	0	0	0	6	0	0
1650	0	0	0	0	0	0	0	0	0	0	6	0
1651	0	0	0	0	0	0	0	0	0	0	0	0
1652	0	0	0	0	0	0	0	0	0	0	0	0
1653	0	0	0	0	0	0	0	0	0	0	6	0
1654	0	0	0	0	0	0	0	0	0	0	6	0
1655	0	0	0	0	0	0	0	0	0	0	6	0
1656	0	0	0	0	0	0	0	0	0	0	6	0
1657	0	0	0	0	0	0	0	0	0	0	6	0
1658	0	0	0	0	0	0	0	0	0	0	0	0
1659	0	0	0	0	0	0	0	0	0	0	6	0
1660	0	0	0	0	0	0	0	0	0	0	0	0
1661	0	0	0	0	0	0	0	0	0	0	6	0
1662	0	0	0	0	0	0	0	0	0	0	6	0
1663	0	0	0	0	0	0	0	0	0	0	6	0
1664	0	0	0	0	0	0	0	0	0	0	6	0
1665	0	0	0	0	0	0	0	0	0	0	6	0

OTU Number	7.11.15 Rep1	7.11.15 Rep2	7.11.15 Rep3	8.29.15 Rep1	8.29.15 Rep2	8.29.15 Rep3	2.6.16 Rep1	2.6.16 Rep2	2.6.16 Rep3	12.10.16 Rep1	12.10.16 Rep2	12.10.16 Rep3
1666	0	0	0	0	0	0	0	0	0	0	6	0
1667	0	0	0	0	0	0	0	0	0	0	6	0
1668	0	0	0	0	0	0	0	0	0	0	6	0
1669	0	0	0	0	0	0	0	0	0	0	0	0
1670	0	0	0	0	0	0	0	0	0	0	0	0
1671	0	0	0	0	0	0	0	0	0	0	0	0
1672	0	0	0	0	0	0	0	0	0	0	0	0
1673	0	0	0	0	0	0	0	0	0	0	0	0
1674	0	0	0	0	0	0	0	0	0	0	0	0
1675	0	0	0	0	0	0	0	0	0	0	0	0
1676	0	0	0	0	0	0	0	0	0	0	6	0
1677	0	0	0	0	0	0	0	0	0	0	6	0
1678	0	0	0	0	0	0	0	0	0	0	0	0
1679	0	0	0	0	0	0	0	0	0	0	0	0
1680	0	0	0	0	0	0	6	0	0	0	0	0
1681	0	0	0	0	0	0	0	0	0	0	0	0
1682	0	0	0	0	0	0	0	0	0	0	6	0
1683	0	0	0	0	0	0	0	0	0	0	0	0
1684	0	0	0	0	0	0	0	0	0	0	6	0
1685	0	0	0	0	0	0	0	0	0	0	0	0
1686	1	0	0	0	2	1	0	0	0	0	0	0
1687	2	0	2	0	1	1	0	0	0	0	0	0
1688	0	0	0	0	0	0	0	0	0	0	6	0
1689	0	0	0	0	0	0	0	0	0	0	0	0
1690	0	0	0	0	0	0	0	0	0	6	0	0
1691	0	0	0	0	0	0	0	0	0	0	0	0
1692	0	0	0	0	0	0	0	0	0	6	0	0
1693	0	0	0	0	0	0	0	0	0	6	0	0
1694	0	0	0	0	0	0	0	0	0	0	6	0
1695	0	0	0	0	0	0	0	0	0	0	0	0
1696	0	0	0	0	0	0	0	0	0	6	0	0
1697	0	0	0	0	0	0	0	0	0	6	0	0
1698	0	0	0	0	0	0	0	0	0	0	0	0
1699	0	0	0	0	0	0	0	0	0	0	6	0
1700	0	0	0	0	0	0	0	0	0	0	6	0
1701	0	0	0	0	0	0	0	0	0	0	0	0
1702	0	0	0	0	0	0	0	0	0	0	0	0
1703	0	0	0	0	0	0	0	0	0	6	0	0
1704	0	0	0	0	0	0	0	0	0	0	0	0
1705	0	0	0	0	0	0	0	0	0	0	0	0
1706	0	0	0	0	0	0	0	0	0	0	0	0
1707	0	0	0	0	0	0	0	0	0	6	0	0
1708	0	0	0	0	0	0	0	0	0	0	6	0
1709	0	0	0	0	0	0	0	0	0	0	0	0
1710	0	0	0	0	0	0	0	0	0	0	0	0

OTU Number	7.11.15 Rep1	7.11.15 Rep2	7.11.15 Rep3	8.29.15 Rep1	8.29.15 Rep2	8.29.15 Rep3	2.6.16 Rep1	2.6.16 Rep2	2.6.16 Rep3	12.10.16 Rep1	12.10.16 Rep2	12.10.16 Rep3
1711	0	0	0	0	0	0	0	0	0	6	0	0
1712	0	0	0	0	0	0	0	0	0	6	0	0
1713	0	0	0	0	0	0	0	0	0	6	0	0
1714	0	0	0	0	0	0	0	0	0	0	0	0
1715	0	0	0	0	0	0	0	0	0	0	6	0
1716	0	0	0	0	0	0	1	0	0	0	0	0
1717	0	0	0	0	0	0	0	0	0	0	0	0
1718	0	0	0	0	0	0	0	0	0	6	0	0
1719	0	0	0	0	0	0	0	0	0	6	0	0
1720	0	0	0	0	0	0	0	0	0	6	0	0
1721	0	0	0	0	0	0	0	0	0	0	0	5
1722	0	0	0	0	0	0	0	0	0	0	0	0
1723	0	0	0	0	0	0	0	0	0	0	0	5
1724	0	0	0	0	0	0	0	0	5	0	0	0
1725	0	0	0	0	0	0	0	0	0	0	0	5
1726	0	0	0	0	0	0	0	0	0	0	5	0
1727	0	0	0	0	0	0	0	0	0	0	0	0
1728	0	0	0	0	0	0	0	0	0	0	0	0
1729	0	0	0	0	0	0	0	0	0	0	0	5
1730	0	0	0	0	0	0	0	0	0	0	0	0
1731	0	0	0	0	0	0	0	0	0	0	0	5
1732	0	0	0	0	0	0	0	0	0	0	5	0
1733	0	0	0	0	0	0	0	0	0	0	5	0
1734	0	0	0	0	0	0	0	0	5	0	0	0
1735	0	0	0	0	0	0	0	0	0	0	5	0
1736	0	0	0	0	0	0	0	0	0	0	0	0
1737	0	0	0	0	0	0	0	0	0	0	5	0
1738	0	0	0	0	0	0	0	0	5	0	0	0
1739	0	0	0	0	0	0	0	0	0	0	0	0
1740	0	0	0	0	0	0	0	0	0	0	0	5
1741	0	0	0	0	0	0	0	0	0	0	5	0
1742	0	0	0	0	0	0	0	0	0	0	0	0
1743	0	0	0	0	0	0	0	0	0	0	0	0
1744	0	0	0	0	0	0	0	0	0	0	0	0
1745	0	0	0	0	0	0	0	0	0	0	0	0
1746	0	0	0	0	0	0	0	0	0	0	0	0
1747	0	0	0	0	0	0	0	0	0	0	0	0
1748	0	0	0	0	0	0	0	0	0	0	0	5
1749	0	0	0	0	0	0	0	0	0	0	0	5
1750	0	0	0	0	0	0	0	0	0	0	0	5
1751	0	0	0	0	0	0	0	0	0	0	0	0
1752	0	0	0	0	0	0	0	0	0	0	0	5
1753	0	0	0	0	0	0	0	0	0	0	5	0
1754	0	0	0	0	0	0	0	0	0	0	0	5
1755	0	0	0	0	0	0	0	0	0	0	0	5

OTU Number	7.11.15 Rep1	7.11.15 Rep2	7.11.15 Rep3	8.29.15 Rep1	8.29.15 Rep2	8.29.15 Rep3	2.6.16 Rep1	2.6.16 Rep2	2.6.16 Rep3	12.10.16 Rep1	12.10.16 Rep2	12.10.16 Rep3
1756	0	0	0	0	0	0	0	0	0	0	0	5
1757	0	0	0	0	0	0	0	0	0	0	0	0
1758	0	0	0	0	0	0	0	0	0	0	5	0
1759	0	0	0	0	0	0	0	0	0	0	0	5
1760	0	0	0	0	0	0	0	0	0	0	0	5
1761	0	0	0	0	0	0	0	0	5	0	0	0
1762	0	0	0	0	0	0	0	0	0	0	0	5
1763	0	0	0	0	0	0	0	0	0	0	5	0
1764	0	0	0	0	0	0	0	0	0	0	0	5
1765	0	0	0	0	0	0	0	0	0	0	0	5
1766	0	0	0	0	0	0	0	0	0	0	0	0
1767	0	0	0	0	0	0	0	0	0	0	0	5
1768	0	0	0	0	0	0	0	0	0	0	0	5
1769	0	0	0	0	0	0	0	0	5	0	0	0
1770	0	0	0	0	0	0	0	0	0	0	5	0
1771	0	0	0	0	0	0	0	0	0	0	5	0
1772	0	0	0	0	0	0	0	0	0	0	0	0
1773	0	0	0	0	0	0	0	0	0	0	0	0
1774	0	0	0	0	0	0	0	0	5	0	0	0
1775	0	0	0	0	0	0	0	0	5	0	0	0
1776	0	0	0	0	0	0	0	0	0	0	0	0
1777	0	0	0	0	0	0	0	0	0	0	0	0
1778	0	0	0	0	0	0	0	0	0	0	0	0
1779	0	0	0	0	0	0	0	0	0	0	0	5
1780	0	0	0	0	0	0	0	0	0	0	0	5
1781	0	0	0	0	0	0	0	0	5	0	0	0
1782	0	0	0	0	0	0	0	0	0	0	0	5
1783	0	0	0	0	0	0	0	0	0	0	0	5
1784	0	0	0	0	0	0	0	0	0	0	0	5
1785	0	0	0	0	0	0	0	0	0	0	0	5
1786	0	0	0	0	0	0	0	0	5	0	0	0
1787	0	0	0	0	0	0	0	0	0	0	0	0
1788	0	0	0	0	0	0	0	0	0	0	0	5
1789	0	0	0	0	0	0	0	0	0	0	0	5
1790	0	0	0	0	0	0	0	0	5	0	0	0
1791	0	2	0	0	1	1	0	0	0	0	0	0
1792	0	0	0	0	0	0	0	0	0	0	0	5
1793	0	1	1	0	2	0	0	0	0	0	0	0
1794	0	0	0	0	0	0	0	0	0	0	0	0
1795	1	1	2	0	1	0	0	0	0	0	0	0
1796	0	0	0	1	0	1	0	0	0	0	0	0
1797	0	0	0	0	0	0	0	0	0	0	0	5
1798	0	0	0	0	0	0	0	0	0	0	5	0
1799	1	0	3	0	0	0	0	0	0	0	0	0
1800	0	0	0	0	0	0	0	0	0	0	0	0

OTU Number	7.11.15 Rep1	7.11.15 Rep2	7.11.15 Rep3	8.29.15 Rep1	8.29.15 Rep2	8.29.15 Rep3	2.6.16 Rep1	2.6.16 Rep2	2.6.16 Rep3	12.10.16 Rep1	12.10.16 Rep2	12.10.16 Rep3
1801	0	0	0	0	0	0	0	0	0	0	0	0
1802	0	0	0	0	0	0	0	0	0	0	0	0
1803	0	0	0	0	4	0	0	0	0	0	0	0
1804	0	0	0	0	0	0	0	5	0	0	0	0
1805	0	0	0	0	0	0	0	0	0	0	5	0
1806	2	0	0	0	0	0	0	0	0	0	0	0
1807	0	0	0	1	0	0	0	0	0	0	0	0
1808	2	0	0	2	1	0	0	0	0	0	0	0
1809	0	0	0	0	0	2	0	0	0	0	0	0
1810	0	0	0	0	1	0	0	0	0	0	0	0
1811	0	0	0	0	0	0	0	0	0	0	0	5
1812	0	0	0	0	0	0	0	0	5	0	0	0
1813	0	0	0	0	0	0	0	0	0	0	0	0
1814	0	0	0	0	0	0	0	0	5	0	0	0
1815	0	0	0	0	0	0	0	0	0	0	0	0
1816	0	0	0	0	0	0	0	0	0	0	0	5
1817	0	0	0	0	0	0	0	0	0	0	0	5
1818	0	0	0	0	0	0	0	0	5	0	0	0
1819	0	0	0	0	0	0	0	0	5	0	0	0
1820	0	0	0	0	0	0	0	0	5	0	0	0
1821	0	1	2	0	1	0	0	0	0	0	0	0
1822	0	0	0	0	0	0	0	0	0	0	0	0
1823	0	0	0	0	0	0	0	0	0	0	5	0
1824	0	0	0	0	0	0	0	0	0	0	0	5
1825	0	0	0	0	0	0	0	0	0	0	0	0
1826	0	0	5	0	0	0	0	0	0	0	0	0
1827	0	1	2	0	0	0	0	0	0	0	0	0
1828	0	0	0	0	0	0	0	0	5	0	0	0
1829	0	0	0	0	0	0	0	0	0	0	0	0
1830	0	0	0	0	0	0	0	0	0	0	0	5
1831	0	0	0	0	0	0	0	0	0	0	0	0
1832	0	0	0	0	0	0	0	0	0	0	5	0
1833	0	0	0	0	0	0	5	0	0	0	0	0
1834	0	0	0	0	0	0	0	0	0	0	0	0
1835	0	0	0	0	0	0	0	0	5	0	0	0
1836	0	0	0	0	0	0	0	0	5	0	0	0
1837	0	0	0	0	0	0	0	0	0	0	0	5
1838	0	0	0	0	0	0	0	0	5	0	0	0
1839	0	0	0	0	0	0	0	0	5	0	0	0
1840	0	0	0	0	0	0	0	0	0	0	0	0
1841	0	0	0	0	0	0	0	0	5	0	0	0
1842	0	0	0	0	0	0	0	0	5	0	0	0
1843	1	0	0	0	0	0	1	0	0	0	0	0
1844	0	0	0	0	0	0	0	0	5	0	0	0
1845	0	0	0	0	0	0	0	0	0	0	0	0

OTU Number	7.11.15 Rep1	7.11.15 Rep2	7.11.15 Rep3	8.29.15 Rep1	8.29.15 Rep2	8.29.15 Rep3	2.6.16 Rep1	2.6.16 Rep2	2.6.16 Rep3	12.10.16 Rep1	12.10.16 Rep2	12.10.16 Rep3
1846	0	0	0	0	0	0	1	0	0	0	0	0
1847	0	0	0	0	0	0	0	0	0	0	0	0
1848	0	0	0	0	0	0	0	0	0	0	5	0
1849	0	0	0	0	0	0	0	0	0	0	5	0
1850	0	0	0	0	0	0	0	0	0	0	0	0
1851	0	0	0	0	0	1	0	0	0	0	0	0
1852	0	0	0	2	0	2	0	0	0	0	0	0
1853	0	0	0	1	1	2	0	0	0	0	0	0
1854	1	0	3	0	0	1	0	0	0	0	0	0
1855	0	0	0	0	0	0	0	0	0	0	0	0
1856	0	0	0	0	0	0	0	0	0	0	5	0
1857	0	0	0	0	0	0	0	0	0	0	0	0
1858	0	0	0	0	0	0	0	0	0	0	0	0
1859	0	0	0	0	0	0	0	0	0	0	5	0
1860	0	0	0	0	0	0	0	0	5	0	0	0
1861	0	0	0	0	0	0	0	0	0	0	5	0
1862	0	0	0	0	0	0	0	0	0	0	0	0
1863	0	0	0	0	0	0	0	0	0	0	5	0
1864	0	0	0	0	0	0	0	0	0	0	0	0
1865	0	0	0	0	0	0	0	0	0	0	0	0
1866	0	0	0	0	0	0	0	0	0	0	0	0
1867	0	0	0	0	0	0	0	0	0	0	0	0
1868	0	0	0	0	0	0	0	0	0	5	0	0
1869	0	0	0	0	0	0	0	0	0	5	0	0
1870	0	0	0	0	0	0	0	0	0	5	0	0
1871	0	0	0	0	0	0	0	0	0	5	0	0
1872	0	0	0	0	0	0	0	0	0	0	0	0
1873	0	0	0	0	0	0	0	0	0	0	0	0
1874	0	0	0	0	0	0	0	0	0	0	0	0
1875	0	0	0	0	0	0	0	5	0	0	0	0
1876	0	0	0	0	0	0	0	0	0	5	0	0
1877	0	0	0	0	0	0	0	0	0	5	0	0
1878	0	0	0	0	0	0	0	0	0	5	0	0
1879	0	0	0	0	0	0	0	5	0	0	0	0
1880	0	0	0	0	0	0	0	0	0	5	0	0
1881	0	0	0	0	0	0	0	0	0	5	0	0
1882	0	0	0	0	0	0	0	0	0	5	0	0
1883	0	0	0	0	0	0	0	0	0	0	0	0
1884	0	0	0	0	0	0	0	0	0	0	0	0
1885	0	0	0	0	0	0	0	0	0	0	0	0
1886	0	0	0	0	0	0	0	0	0	0	0	0
1887	0	0	0	0	0	0	0	5	0	0	0	0
1888	0	0	0	0	0	0	0	0	0	5	0	0
1889	0	0	0	0	0	0	0	0	0	0	0	0
1890	1	1	0	0	0	0	0	0	0	0	0	0

OTU Number	7.11.15 Rep1	7.11.15 Rep2	7.11.15 Rep3	8.29.15 Rep1	8.29.15 Rep2	8.29.15 Rep3	2.6.16 Rep1	2.6.16 Rep2	2.6.16 Rep3	12.10.16 Rep1	12.10.16 Rep2	12.10.16 Rep3
1891	0	0	0	0	0	0	0	0	0	5	0	0
1892	0	0	0	0	0	0	0	0	0	5	0	0
1893	0	0	0	0	0	0	0	0	0	5	0	0
1894	0	0	0	0	0	0	0	5	0	0	0	0
1895	0	0	0	0	0	0	0	0	0	5	0	0
1896	0	0	0	0	0	0	0	0	0	0	5	0
1897	0	0	0	0	0	0	0	0	0	0	5	0
1898	0	0	0	0	0	0	0	0	0	0	0	0
1899	0	0	0	0	0	0	0	0	0	0	5	0
1900	0	0	0	0	0	0	0	0	0	0	0	0
1901	0	0	0	0	0	0	0	0	0	0	5	0
1902	0	0	0	0	0	0	0	0	0	0	0	0
1903	0	0	0	0	0	0	0	0	0	5	0	0
1904	0	0	0	0	0	0	0	5	0	0	0	0
1905	0	0	0	0	0	0	0	0	0	5	0	0
1906	0	0	0	0	0	0	0	5	0	0	0	0
1907	0	0	0	0	0	0	0	0	0	5	0	0
1908	0	0	0	0	0	0	0	0	0	5	0	0
1909	0	0	0	0	0	0	0	0	0	0	0	0
1910	0	0	0	0	0	0	0	0	0	0	0	0
1911	0	5	0	0	0	0	0	0	0	0	0	0
1912	0	0	0	0	0	0	0	0	0	0	0	0
1913	0	0	0	0	0	0	0	0	0	5	0	0
1914	0	0	0	0	0	0	0	0	0	5	0	0
1915	0	0	0	0	0	0	0	0	0	5	0	0
1916	0	0	0	0	0	0	0	0	0	5	0	0
1917	0	0	0	0	0	0	0	0	0	5	0	0
1918	0	0	0	0	0	0	1	0	0	0	0	0
1919	0	0	0	0	0	0	0	0	0	5	0	0
1920	0	0	0	0	0	0	0	0	0	5	0	0
1921	0	0	0	0	0	0	0	0	0	0	0	0
1922	0	0	0	0	0	0	0	0	0	5	0	0
1923	0	0	0	0	0	0	0	0	0	5	0	0
1924	0	0	0	0	0	0	5	0	0	0	0	0
1925	0	0	0	0	0	0	0	5	0	0	0	0
1926	0	0	0	0	0	0	0	5	0	0	0	0
1927	0	0	0	0	0	0	0	0	0	0	0	0
1928	0	0	0	0	0	0	0	5	0	0	0	0
1929	0	0	0	0	0	0	0	0	0	0	0	0
1930	0	0	0	0	0	0	0	5	0	0	0	0
1931	0	0	0	0	0	0	0	0	0	5	0	0
1932	0	0	0	0	0	0	0	0	0	5	0	0
1933	0	0	0	0	0	0	0	5	0	0	0	0
1934	0	0	0	0	0	0	0	0	0	0	0	0
1935	0	0	0	0	0	0	0	0	0	5	0	0

OTU Number	7.11.15 Rep1	7.11.15 Rep2	7.11.15 Rep3	8.29.15 Rep1	8.29.15 Rep2	8.29.15 Rep3	2.6.16 Rep1	2.6.16 Rep2	2.6.16 Rep3	12.10.16 Rep1	12.10.16 Rep2	12.10.16 Rep3
1936	2	1	0	0	1	0	0	0	0	0	0	0
1937	1	1	0	1	0	0	0	0	0	0	0	0
1938	4	0	0	0	0	0	0	0	0	0	0	0
1939	0	0	0	0	0	0	0	0	0	5	0	0
1940	0	0	0	0	0	0	0	5	0	0	0	0
1941	0	0	0	0	0	0	0	5	0	0	0	0
1942	0	0	0	0	0	0	0	5	0	0	0	0
1943	0	0	0	0	0	0	0	0	0	0	0	0
1944	0	0	0	0	0	0	0	0	0	5	0	0
1945	0	0	0	0	0	0	0	0	0	5	0	0
1946	0	0	0	0	0	0	0	0	0	5	0	0
1947	0	0	0	0	0	0	0	0	0	5	0	0
1948	0	0	0	0	0	0	0	0	0	5	0	0
1949	0	0	0	0	0	0	0	0	0	5	0	0
1950	0	0	0	0	0	0	0	0	0	5	0	0
1951	0	0	0	0	0	0	0	0	0	0	0	0
1952	0	0	0	0	0	0	5	0	0	0	0	0
1953	0	0	0	0	0	0	0	5	0	0	0	0
1954	0	0	0	0	0	0	0	5	0	0	0	0
1955	0	0	0	0	0	0	0	0	0	5	0	0
1956	0	0	0	0	0	0	0	5	0	0	0	0
1957	0	0	0	0	0	0	0	5	0	0	0	0
1958	0	0	0	0	0	0	0	5	0	0	0	0
1959	0	0	0	0	0	0	5	0	0	0	0	0
1960	0	0	0	0	0	0	0	0	0	5	0	0
1961	0	1	0	0	0	0	0	0	0	0	0	0
1962	0	0	0	0	0	0	0	5	0	0	0	0
1963	0	0	0	0	0	0	0	5	0	0	0	0
1964	0	0	0	0	0	0	0	0	0	5	0	0
1965	0	0	0	0	0	0	0	5	0	0	0	0
1966	0	0	0	0	0	0	0	0	0	5	0	0
1967	0	0	0	0	0	0	0	0	0	5	0	0
1968	0	0	0	0	0	0	0	5	0	0	0	0
1969	0	0	0	0	0	0	0	0	0	0	0	0
1970	0	0	0	0	0	0	0	0	0	0	5	0
1971	0	0	0	0	1	0	0	0	0	0	0	0
1972	0	0	0	0	0	0	0	0	0	0	0	0
1973	0	0	0	0	0	0	0	0	0	0	0	0
1974	0	0	0	0	0	0	0	0	0	0	5	0
1975	0	0	0	0	0	0	0	0	0	0	0	0
1976	0	0	0	0	0	0	0	0	0	0	0	0
1977	0	0	0	0	0	0	0	0	0	0	0	0
1978	0	0	0	0	0	0	0	0	0	0	0	0
1979	0	0	0	0	0	0	0	0	0	0	0	0
1980	0	0	0	0	0	0	0	0	0	0	0	0

OTU Number	7.11.15 Rep1	7.11.15 Rep2	7.11.15 Rep3	8.29.15 Rep1	8.29.15 Rep2	8.29.15 Rep3	2.6.16 Rep1	2.6.16 Rep2	2.6.16 Rep3	12.10.16 Rep1	12.10.16 Rep2	12.10.16 Rep3
1981	0	0	0	0	0	0	0	0	0	0	0	0
1982	0	0	0	0	0	0	0	0	0	0	5	0
1983	0	0	0	1	1	0	0	0	0	0	0	0
1984	0	0	1	0	0	3	0	0	0	0	0	0
1985	1	0	0	0	2	1	0	0	0	0	0	0
1986	1	0	1	0	1	1	0	0	0	0	0	0
1987	0	0	0	0	0	1	0	0	0	0	0	0
1988	0	0	0	0	0	1	0	0	0	0	0	0
1989	0	1	0	1	1	0	0	0	0	0	0	0
1990	1	0	0	1	0	0	0	0	0	0	0	0
1991	1	0	0	0	0	0	0	0	0	0	0	0
1992	3	0	0	1	1	0	0	0	0	0	0	0
1993	0	0	0	0	0	0	0	0	0	0	0	0
1994	0	0	0	0	0	0	0	0	0	0	0	0
1995	0	0	0	0	0	0	0	0	0	0	5	0
1996	0	0	0	0	0	0	0	0	0	0	0	5
1997	0	0	0	0	0	0	0	0	0	0	0	5
1998	0	0	0	0	0	0	0	0	0	0	5	0
1999	0	0	0	0	0	0	0	0	0	0	0	5
2000	0	0	0	0	0	0	0	0	0	0	0	5
